# Early detection of exon 1 huntingtin aggregation in zQ175 brains by molecular and histological approaches

**DOI:** 10.1093/braincomms/fcad010

**Published:** 2023-01-20

**Authors:** Edward J Smith, Kirupa Sathasivam, Christian Landles, Georgina F Osborne, Michael A Mason, Casandra Gomez-Paredes, Bridget A Taxy, Rebecca E Milton, Anne Ast, Franziska Schindler, Chuangchuang Zhang, Wenzhen Duan, Erich E Wanker, Gillian P Bates

**Affiliations:** Huntington’s Disease Centre, Department of Neurodegenerative Disease and UK Dementia Research Institute at UCL, Queen Square Institute of Neurology, University College London, London WC1N 3BG, UK; Huntington’s Disease Centre, Department of Neurodegenerative Disease and UK Dementia Research Institute at UCL, Queen Square Institute of Neurology, University College London, London WC1N 3BG, UK; Huntington’s Disease Centre, Department of Neurodegenerative Disease and UK Dementia Research Institute at UCL, Queen Square Institute of Neurology, University College London, London WC1N 3BG, UK; Huntington’s Disease Centre, Department of Neurodegenerative Disease and UK Dementia Research Institute at UCL, Queen Square Institute of Neurology, University College London, London WC1N 3BG, UK; Huntington’s Disease Centre, Department of Neurodegenerative Disease and UK Dementia Research Institute at UCL, Queen Square Institute of Neurology, University College London, London WC1N 3BG, UK; Huntington’s Disease Centre, Department of Neurodegenerative Disease and UK Dementia Research Institute at UCL, Queen Square Institute of Neurology, University College London, London WC1N 3BG, UK; Huntington’s Disease Centre, Department of Neurodegenerative Disease and UK Dementia Research Institute at UCL, Queen Square Institute of Neurology, University College London, London WC1N 3BG, UK; Huntington’s Disease Centre, Department of Neurodegenerative Disease and UK Dementia Research Institute at UCL, Queen Square Institute of Neurology, University College London, London WC1N 3BG, UK; Neuroproteomics, Max Delbrück Center for Molecular Medicine in the Helmholtz Association, Berlin 13125, Germany; Neuroproteomics, Max Delbrück Center for Molecular Medicine in the Helmholtz Association, Berlin 13125, Germany; Division of Neurobiology, Department Psychiatry and Behavioral Sciences; Department Neuroscience, Johns Hopkins University School of Medicine, Baltimore, MD 21287, USA; Division of Neurobiology, Department Psychiatry and Behavioral Sciences; Department Neuroscience, Johns Hopkins University School of Medicine, Baltimore, MD 21287, USA; Neuroproteomics, Max Delbrück Center for Molecular Medicine in the Helmholtz Association, Berlin 13125, Germany; Huntington’s Disease Centre, Department of Neurodegenerative Disease and UK Dementia Research Institute at UCL, Queen Square Institute of Neurology, University College London, London WC1N 3BG, UK

**Keywords:** Huntington’s disease, huntingtin HTRF bioassay, huntingtin aggregation, zQ175 knock-in mouse model, HTTexon1

## Abstract

Huntingtin-lowering approaches that target huntingtin expression are a major focus for therapeutic intervention for Huntington’s disease. When the cytosine, adenine and guanine repeat is expanded, the huntingtin pre-mRNA is alternatively processed to generate the full-length huntingtin and *HTT1a* transcripts. *HTT1a* encodes the aggregation-prone and highly pathogenic exon 1 huntingtin protein. In evaluating huntingtin-lowering approaches, understanding how the targeting strategy modulates levels of both transcripts and the huntingtin protein isoforms that they encode will be essential. Given the aggregation-propensity of exon 1 huntingtin, the impact of a given strategy on the levels and subcellular location of aggregated huntingtin will need to be determined. We have developed and applied sensitive molecular approaches to monitor the levels of aggregated and soluble huntingtin isoforms in tissue lysates. We have used these, in combination with immunohistochemistry, to map the appearance and accumulation of aggregated huntingtin throughout the CNS of zQ175 mice, a model of Huntington’s disease frequently chosen for preclinical studies. Aggregation analyses were performed on tissues from zQ175 and wild-type mice at monthly intervals from 1 to 6 months of age. We developed three homogeneous time-resolved fluorescence assays to track the accumulation of aggregated huntingtin and showed that two of these were specific for the exon 1 huntingtin protein. Collectively, the homogeneous time-resolved fluorescence assays detected huntingtin aggregation in the 10 zQ175 CNS regions by 1–2 months of age. Immunohistochemistry with the polyclonal S830 anti-huntingtin antibody showed that nuclear huntingtin aggregation, in the form of a diffuse nuclear immunostain, could be visualized in the striatum, hippocampal CA1 region and layer IV of the somatosensory cortex by 2 months. That this diffuse nuclear immunostain represented aggregated huntingtin was confirmed by immunohistochemistry with a polyglutamine-specific antibody, which required formic acid antigen retrieval to expose its epitope. By 6 months of age, nuclear and cytoplasmic inclusions were widely distributed throughout the brain. Homogeneous time-resolved fluorescence analysis showed that the comparative levels of soluble exon 1 huntingtin between CNS regions correlated with those for huntingtin aggregation. We found that soluble exon 1 huntingtin levels decreased over the 6-month period, whilst those of soluble full-length mutant huntingtin remained unchanged, data that were confirmed for the cortex by immunoprecipitation and western blotting. These data support the hypothesis that exon 1 huntingtin initiates the aggregation process in knock-in mouse models and pave the way for a detailed analysis of huntingtin aggregation in response to huntingtin-lowering treatments.

## Teaser

Smith et al. have analysed the temporal and spatial appearance of aggregated HTT in the brains of the zQ175 Huntington’s disease mouse model. They find that the mutant exon 1 HTT protein initiates aggregation and that this can be detected throughout the CNS by one to 2 months of age.

## Graphical Abstract

**Graphical abstract:**
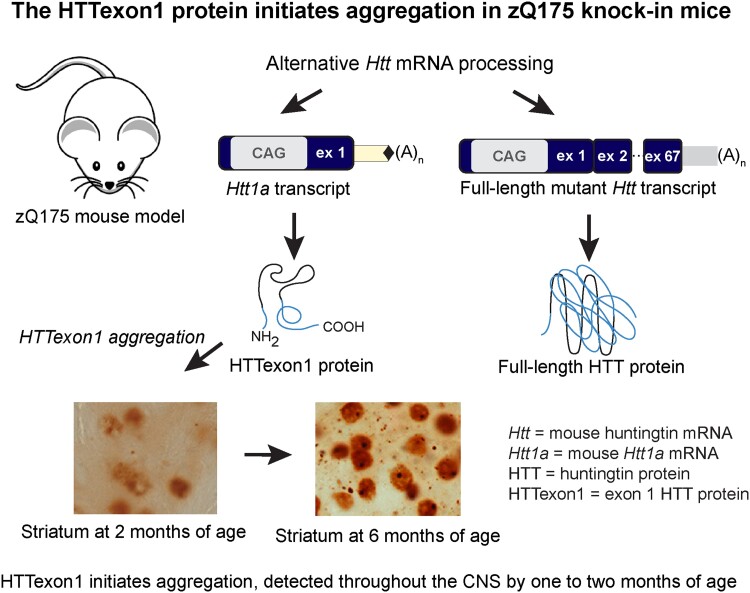

## Introduction

Huntington’s disease is an inherited neurodegenerative disorder that manifests with psychiatric disturbances, movement disorders and cognitive decline.^1^ It is caused by a cytosine, adenine and guanine (CAG) repeat expansion in exon 1 of the huntingtin (*HTT*) gene that encodes a polyglutamine (polyQ) tract in the huntingtin protein (HTT). Individuals with repeats of 35 CAGs remain unaffected whereas repeats of 40 CAGs and above are fully penetrant and will result in the onset of disease within a normal lifespan. The age at onset is determined by the length of the CAG repeat, together with genetic and environmental modifiers,^2^ and CAG expansions of approximately 65 and above cause juvenile Huntington’s disease with onset in childhood or adolescence.^3^ PolyQ inclusions are deposited in the brains of individuals with Huntington’s disease^4,5^ and cell loss occurs in the striatum, cortex and other brain regions.^6,7^

The CAG repeat is unstable in both the germline and somatic tissues,^8,9^ with very large CAG repeat expansions having been identified in post-mortem brains from patients with Huntington’s disease.^10^ It was known from studies with mouse models of Huntington’s disease that the ablation of specific DNA mismatch repair genes attenuated somatic CAG instability.^11–13^ Therefore, the demonstration that genetic modifiers for Huntington’s disease result from variants in mismatch, and other DNA repair genes,^14–17^ has suggested that somatic CAG repeat expansion drives the age of onset and rate of disease progression. The *HTT* gene contains 67 exons, and in the context of an expanded CAG repeat, the *HTT* mRNA can be alternatively processed, activating polyadenylation (polyA) sites within intron 1 to generate the *HTT1a* transcript.^18,19^*HTT1a* encodes the exon 1 HTT protein (HTTexon1), which is known to be highly aggregation-prone^20^ and pathogenic.^21^ As the alternative processing of *HTT* mRNA, and consequently, the levels of *HTT1a* and HTTexon1 increase with increasing CAG repeat length,^19^ HTTexon1 may be the pathogenic effector of somatic CAG repeat expansion in Huntington’s disease.

There are currently no disease-modifying therapies for Huntington’s disease and strategies that aim to lower the levels of the HTT protein hold great promise.^22^ Transcriptional and post-transcriptional approaches that include small interfering RNAs,^23^ microRNAs,^24^ antisense oligonucleotides,^25^ zinc-finger proteins^26^ and small molecule splicing modulators^27^ are in clinical development.^28^ These modalities either target the full-length *HTT* transcript or both *HTT* and *HTT1a*. However, the comparative merits of targeting full-length *HTT* or *HTT1a*, and whether one or both *HTT* transcripts should be lowered is not known. The zQ175 knock-in mouse model of Huntington’s disease^29,30^ is frequently used for the target validation and preclinical screening of *HTT*-lowering approaches.^26^ These mice were generated by replacing exon 1 of mouse *Htt* with exon 1 from human *HTT*, carrying a highly expanded CAG repeat^30,31^ from which the neo-selectable marker has been removed (delta neo).^32,33^ This model can be used to compare the efficacy of targeting full-length *Htt*, *Htt1a* or both, as well as to determine the most effective timing of treatment initiation relative to phenotype onset and progression.

Given that the level of HTTexon1 production is related to CAG repeat expansion, and that HTTexon1 is a very aggregation-prone protein,^20^ it is important that the accumulation of HTT aggregation throughout the brains of zQ175 mice has been defined and that this can be quantified. To better inform HTT-lowering experiments we have further developed and optimized molecular and immunohistochemical techniques to document the temporal and spatial appearance of aggregated HTT in zQ175 CNS regions between 1 and 6 months of age. We found that aggregated HTT could be detected and quantified in all regions of the brain by 1–2 months of age using molecular approaches. In all cases, this could first be visualized by immunohistochemistry as diffusely distributed staining throughout the nucleus and was apparent in the striatum by 6 weeks of age and in regions of the cortex and hippocampus by 2 months. Overall, the levels of soluble HTTexon1 decreased with the accumulation of aggregated HTT, whilst over this time frame, the level of full-length HTT remained constant. These data are consistent with mutant versions of the HTTexon1 protein initiating the aggregation process.

## Materials and methods

### Mouse breeding and maintenance

All procedures were performed in accordance with the Animals (Scientific Procedures) Act 1986 and were approved by the University College London Animal Welfare and Ethical Review Body Committee. Heterozygous zQ175 mice were either bred in-house by backcrossing males to C57BL/6J females (Charles River) or obtained from the CHDI Foundation colony at the Jackson Laboratory (Bar Harbor, Maine) on a C57BL/6J background.

Mouse husbandry and health monitoring were as previously described.^34^ Within each colony, genetically modified and wild-type mice were group housed with up to five mice per cage, dependent on gender, but genotypes were mixed. Mice were housed in individually ventilated cages with Aspen Chips 4 Premium bedding (Datesand) and environmental enrichment which included chew sticks and a play tunnel (Datesand). They had unrestricted access to food (Teklad global 18% protein diet, Envigo) and water. The temperature was regulated at 21 °C ± 1 °C and animals were kept on a 12 h light/dark cycle. The animal facility was barrier-maintained and quarterly non-sacrificial Federation of European Laboratory Animal Science Associations screens found no evidence of pathogens. For molecular analyses, mice were sacrificed by a schedule 1 procedure, brains were rapidly dissected and tissues were snap-frozen in liquid nitrogen and stored at −80 °C.

Transgenic N171-82Q mice^35^ on a B6C3F1/J hybrid background were bred by backcrossing N171-82Q males to B6C3F1/J females (Jackson Laboratory, Bar Harbor, Maine) and obtained from Wenzhen Duan’s colony at Johns University Hopkins, Baltimore, USA. All procedures were approved by the Institutional Animal Care and Use Committee of Johns Hopkins University.

### Genotyping and CAG repeat sizing

Genotyping and CAG repeat sizing for zQ175 or N171-82Q mice were performed as previously described.^34^ The mean and standard deviation for the CAG repeat-sizes for the zQ175 mice that were used for molecular analyses and immunohistochemistry were 203 ± 9.07 at 1 month, 195 ± 4.20 at 2 months 191 ± 3.84 at 3 months, 192 ± 4.25 at 4 months, 197 ± 6.84 at 5 months and 194 ± 3.77 at 6 months of age. The CAG repeat-sizes for the zQ175 mice used for immunohistochemistry were 206 ± 2.08 at 5 weeks, 203 ± 7.02 at 6 weeks and 203 ± 2.65 at 7 weeks of age. The CAG repeat size in the N171-82Q mouse line is stable.

### Antibodies

The primary and secondary antibodies used for immunohistochemistry, homogeneous time-resolved fluorescence (HTRF), immunoprecipitation and western blotting are summarized in Supplementary Table 1.

### Immunohistochemistry

Mice were transcardially perfusion fixed with 4% paraformaldehyde (Pioneer Research Chemical Ltd) and tissue processing and the storage of sections was as previously documented.^36^ The immunostaining of sections was as previously described^36^ with some minor modifications. Sections were washed in phosphate buffer saline (PBS) before endogenous peroxide activity was quenched using 1% hydrogen peroxide (H_2_O_2_) in PBS for 30 min followed by three washes in PBS. Before immunostaining, non-specific binding was blocked for 1 h using 10% normal goat or rabbit serum (Sigma), as appropriate in PBST (0.3–0.5% TritonX-100 (Sigma) in PBS). Primary antibodies were applied in fresh blocking solution overnight at 4 °C. Sections were washed three times in PBS before application of the biotinylated secondary antibody (1:500, Vector, Peterborough, UK) for 2 h. Sections were washed three times in PBS before signal amplification using Elite ABC reagent (Vector, Peterborough, UK). Biotinylated antibody staining was visualized using FastDAB (0.5 mg/ml) (Sigma) in PBS and activated with H_2_O_2_. Sections were treated with 3.3′-diaminobenzidine (DAB) solution for 40 s and rapidly washed with ice-cold PBS to stop the reaction. The sections were washed a further three times with ice-cold PBS to remove the DAB solution completely before mounting on glass slides. Slides were then processed through 70, 90 and 100% alcohols and cleared in xylene before cover-slipping with DPX Mountant (Sigma).

For staining with cresol violet, sections were mounted onto slides, allowed to air dry for 3–4 h and then transferred to 50 °C for 30 min. The sections were rinsed in water and then cresol violet solution (diluted 1:10 with distilled and deionized H_2_O just before use) was applied for 3 min. The slides were quickly rinsed in two changes of distilled water. The sections were dehydrated via 70, 90 and 100% alcohol, cleared in xylene, mounted in DPX Mountant (Sigma) and allowed to air dry overnight.

The antigen retrieval of polyQ epitopes from polyQ aggregates was as previously described with some minor modifications.^36,37^ Before 4H7H7 or 2B7 primary antibody application, antigen retrieval was used to expose aggregation-buried epitopes. Sections were treated three times with 88% formic acid (Alfa Aesar) for 10 min followed by three washes in PBS. The sections were treated with 0.5 M ethanolamine (Sigma) in PBS (pH 9.5, blocking excess reacting aldehyde sites) for 1 h with the addition of ascorbic acid (EDQM) to a concentration of 5 mM for the final 10 min (reducing Schiff bases). Sections were rinsed in PBS three times and blocked three times with PBST for 10, 30 and 10 min. Biotinylated 4H7H7^37^ (1:3000) was then applied in fresh PBST overnight at 4 °C. Sections were washed three times in PBS before signal amplification using Elite ABC reagent (Vector, Peterborough, UK). Sections were washed three times in TI buffer [0.05 M Tris pH 7.4, 0.05 M Imidazole (Sigma) in PBS]. Signals were amplified using the Tyramide Signal Amplification kit (Perkin Elmer) according to the manufacturer’s recommendations and then washed three times in PBST before a second application of Elite ABC reagent.

Histological images were obtained using a Zeiss AxioSkop2 Plus microscope fitted with a Zeiss AxioCam 208 colour camera. Images were recorded using Zeiss ZEN 3.2 (blue edition). For thresholding, images were converted to eight-bit in ImageJ and a signal intensity level was chosen to indicate staining above the background.

### HTRF

HTRF was performed as previously described using *n* = 6/genotype/age with equal numbers of males and females.^34^ Briefly, individual brain regions were homogenized (w/v) in ice-cold bioassay buffer (PBS, 1% Triton-X-100) supplemented with cOmplete protease inhibitor cocktail tablets (Roche), using a Fast-Prep-24 instrument (MP Biomedicals). Crude lysates were used for HTT aggregation assays, whereas for soluble HTT assays the supernatant was used following centrifugation (3500*×g* for 10 min). For HTRF assay optimizations, antibody concentrations were established by maintaining the terbium donor at 1 ng/well, whilst titrating the d2 acceptor at either 1, 2, 5, 10, 20 or 40 ng/well (Supplementary Figs. 1 and 2). At the same time, a four-point lysate titration was performed (Supplementary Figs. 1 and 2) by diluting zQ175 cortical homogenates with either age-matched wild-type homogenates at 2 months of age for soluble mutant HTT assays or 12-months for HTT aggregation assays. Homogenates at 6 months of age were diluted with lysis buffer for the endogenous mouse HTT and total soluble full-length HTT assays. Antibody and lysate concentrations were chosen which resulted in a titration curve that was as close to linear as possible (Supplementary Table 2).

HTRF assays were performed in triplicate. The donor and acceptor antibodies were added in 5 µL HTRF detection buffer (50 mM Nah_2_PO_4_, 0.2 M KF, 0.1% bovine serum albumin, 0.05% Tween-20) to give the optimized antibody concentration when added to 10 µL of the optimized lysate concentration in a 384-well ProxiPlate (Greiner Bio-One). Following incubation for 3 h on an orbital shaker (250 rpm), plates were read using an EnVision (Perkin Elmer) plate reader as previously described.^34^

### FRET-based aggregate seeding assay

The FRET-based aggregate seeding assay (FRASE) was as previously described.^36^ For brain tissues, a 10% (w/v) total protein lysate was prepared by homogenizing in ice-cold FRASE buffer (10 mM Tris–HCl pH 7.4, 0.8 M NaCl, 1 mM ethylenediaminetetraacetic acid (EDTA), 10% sucrose, 0.25 U/mL benzonase), with complete protease inhibitor tablets (Roche), in Precellys^®^ CK14 lysing kit tubes (Precellys^®^ 24 Homogenizer). The homogenate was incubated for 1 h at 4 °C on a rotating wheel and protein concentration was determined using the Pierce^™^ Bincinchoninic Acid (BCA) protein assay kit (Thermo Fisher Scientific). The FRASE assay was performed and evaluated according to the method previously described^38^ using 2.5 µg of crude lysate per replicate.

### Immunoprecipitation and western blotting

Immunoprecipitations from wild-type and zQ175 cortical samples were performed using the anti-polyglutamine 3B5H10 antibody (Sigma-Aldrich) as described previously.^18,39^ For western blotting, proteins were denatured, separated by 8% sodium dodecyl sulphate (SDS)-polyacrylamide acrylamide gel electrophoresis, blotted onto nitrocellulose membranes and detected by chemiluminescence, as described previously.^36,39^ Primary antibody dilutions were 1:1000, except for MAB5490 which was 1:500. Quantification of western blots was performed using the Image Lab software (Bio-Rad).

### Statistical analysis

Data were screened for outliers using Grubb’s test (GraphPad Prism v9). Statistical analysis was performed with GraphPad Prism (v9) using two-way ANOVA with Bonferroni *post hoc* test. Graphs were prepared using GraphPad Prism (v9). *P*-values <0.05 were considered statistically significant.

## Results

**Figure 1:**
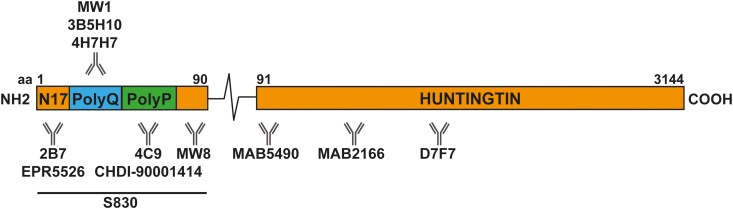
**Schematic showing the location of antibody binding sites on the HTT protein.** The HTT protein is 3144 amino acids long and HTTexon1 extends to amino acid 90. N17 = first 17 amino acids, polyQ = polyglutamine tract, polyP = polyproline repeats and polyproline-rich domain. The relative antibody binding sites are shown. S830 is a polyclonal antibody raised against HTTexon1. aa, amino acids.

We have recently established a series of HTRF assays that can be used to track soluble and aggregated isoforms of the HTT protein. The aggregated isoforms may include oligomers, protofibrils, mature fibrils and inclusions. These assays can detect mutant and wild-type forms of soluble full-length HTT, soluble HTTexon1 and aggregated HTT isoforms.^34^ The location of the antibodies used in these assays is indicated in Fig. 1. Here we have applied these assays to track the levels of soluble and aggregated HTT in 10 CNS regions collected from heterozygous zQ175 knock-in and wild-type mice at monthly intervals from 1 to 6 months of age. zQ175 mice have exon 1 of mouse *Htt* replaced with exon 1 of human *HTT* with repeat expansions of approximately 200 CAGs.

### HTRF assays detect HTT aggregation throughout the zQ175 brain by 1–2 months of age

**Figure 2:**
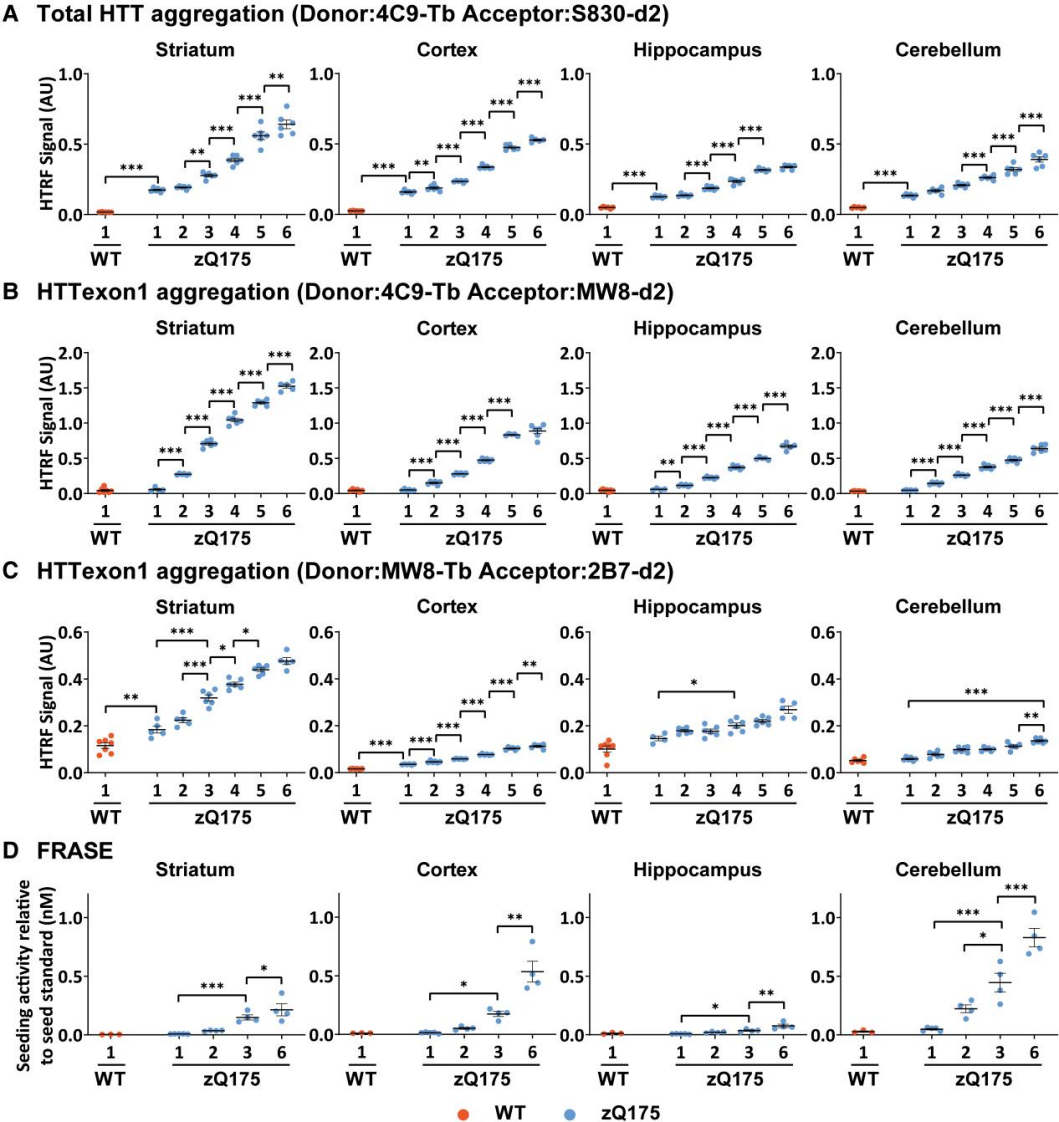
**Comparative accumulation of aggregated HTT in zQ175 brains as measured by three HTRF assays and FRASE. (A–C)** Aggregated HTT as detected by three HTRF assays was first detected in the cortex, striatum, hippocampus and cerebellum of heterozygous zQ175 mice at **(A)** 1 month of age using the 4C9-S830 assay, **(B)** 2 months of age using the 4C9-MW8 assay and **(C)** at 1 month in the striatum and cortex using the MW8-2B7 assay. In all cases, HTT aggregation increased with age with the relative levels between brain regions being comparable for all three assays. The complete HTRF assay data sets for all 10 CNS regions are shown in Supplementary Fig. 3 for 4C9-S830, Supplementary Fig. 4 for 4C9-MW8 and Supplementary Fig. 5 for MW8-2B7 assays (*N* = 6/genotype/age). (**D**) The polymerization of HTT aggregation seeds as measured by the FRASE assay. In contrast to the HTRF assays, the highest signal was obtained in the cerebellum, followed by the cortex and then the striatum and hippocampus (*N* = 4/genotype/age). Error bars are mean ± SEM. The test statistic, degrees of freedom and *P-*values for the two-way ANOVA are provided in Supplementary Tables 3–6. Statistical differences are indicated when there is a difference from 1 month to the next, and to indicate the age at which aggregated HTT can first be detected *P ≤ 0.05, **P ≤ 0.01, ***P ≤ 0.001. WT = wild type. AU, arbitrary units.

We began by comparing the age at which HTT aggregation could first be detected, and the rate at which it increased up to 6 months of age, using the three HTRF HTT aggregation assays: 4C9-S830, 4C9-MW8 and MW8-2B7. Aggregated HTT levels in the striatum, cortex, hippocampus and cerebellum are presented in Fig. 2A–C. The complete sets of results for all 10 CNS regions are shown in the Supplementary material: 4C9-S830 (Supplementary Fig. 3), 4C9-MW8 (Supplementary Fig. 4) and MW8-2B7 (Supplementary Fig. 5).

Aggregated HTT could be detected in all CNS regions using the 4C9-S830 assay and, of these, was first apparent at 1 month of age in all brain regions except for the colliculus, brain stem and spinal cord (Fig. 2A and Supplementary Fig. 3). The highest levels were present in the striatum, followed by the cortex, with the lowest levels in thalamus, brain stem and spinal cord. Aggregated HTT levels continued to increase over the 6-month period in striatum, cortex, cerebellum and olfactory bulb, whereas in other brain regions levels had plateaued between 5 and 6 months of age (Fig. 2A and Supplementary Fig. 3). Aggregated HTT as detected by the 4C9-MW8 assay showed a similar pattern (Fig. 2B and Supplementary Fig. 4) except that aggregated HTT was not detected until 2 months of age in any region (Fig. 2C and Supplementary Fig. 5). The brain regions in which aggregated HTT could be detected by MW8-2B7, were those for which the highest levels were detected using the 4C9-S830 and 4C9-MW8 assays.

In general, the comparative levels of HTT aggregation between brain regions were consistent between the three HTRF assays, with the highest levels detected in the striatum in all cases.

### The profile of aggregate seeding as measured by FRASE differs from that detected by HTRF

The FRASE provides a measure of HTT aggregates capable of seeding polymerization.^38^ To determine when the seeding competence of HTT aggregates could be first detected, we selected striatal, cortical, hippocampal and cerebellar tissues for FRASE as HTT aggregates could be detected in these brain regions by 1–2 months of age using HTRF (Fig. 2D). The FRASE assay was not as sensitive as the HTRF assays to detect HTT aggregation at earlier ages. However, the relative levels of HTT seeds between brain regions differed from HTRF, with the highest levels detected in the cerebellum followed by the cortex, then the striatum and finally the hippocampus.

### The 4C9-MW8 and MW8-2B7 HTRF aggregation assays are specific to HTTexon1

The MW8 antibody was raised against the eight C-terminal amino acids of the HTTexon1 protein.^40^ We have previously shown that on western blots, MW8 is specific for the C-terminus of HTTexon1, which terminates in a proline residue and does not detect this peptide when it is present in full-length HTT or other C-terminal proteolytic fragments that are longer than HTTexon1.^39^ We had also shown that MW8 acts as a neo-epitope antibody for the C-terminus of HTTexon1 by HTRF, meso scale discovery (MSD) and AlphaLISA assays, with the consequence that the 2B7-MW8 assay is specific for soluble HTTexon1 on all three platforms.^34^

**Figure 3:**
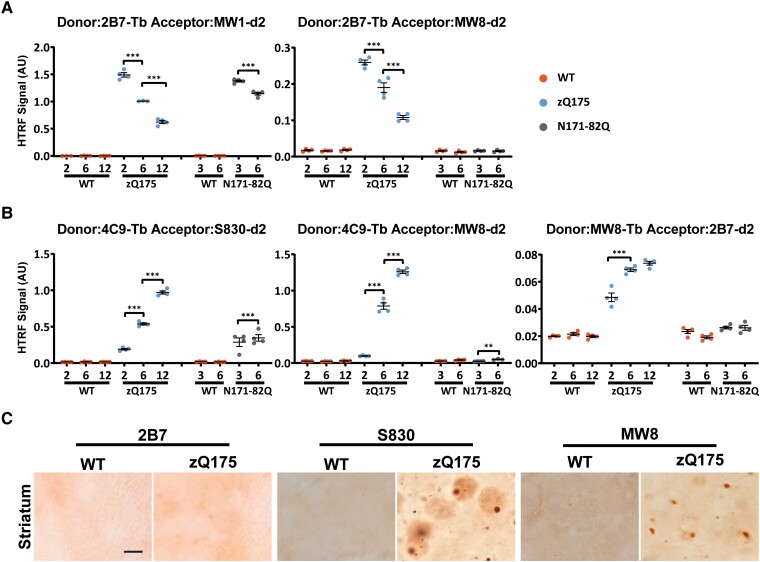
**The 4C9-MW8 and MW8-2B7 HTT aggregation assays are specific for HTTexon1.** (**A**) The 2B7-MW1 and 2B7-MW8 HTRF assays were applied to cortical lysates from heterozygous zQ175 mice and wild-type littermates at 2, 6 and 12 months of age and from N171-82Q mice and their wild-type littermates at 3 and 6 months of age. The 2B7-MW1 assay detects ‘soluble mutant HTT’ and a signal was detected in both the zQ175 and N171-82Q lysates. There was no signal in the wild-type mice as they do not produce mutant HTT. In contrast, the 2B7-MW8 assay only produced a signal in zQ175 lysates, but not those from N171-82Q mice, confirming its specificity for soluble HTTexon1. There was no signal in wild-type mice as they do not produce HTTexon1. *N* = 4/genotype/age. WT = wild type, AU = arbitrary units. (**B**) The three HTRF aggregation assays (4C9-S830, 4C9-MW8 and MW8-2B7) were applied to cortical lysates from zQ175 mice and wild-type littermates at 2, 6 and 12 months of age and from N171-82Q mice and their wild-type littermates at 3 and 6 months of age. The 4C9-S830 assay detected aggregated HTT in both zQ175 and N171-82Q mice, whereas, in contrast, the 4C9-MW8 and MW8-2B7 assays only detected aggregated HTT in zQ175 mice, indicating that these assays are specific for aggregated HTTexon1. *N* = 4/genotype/age. Error bars are mean ± SEM. The test statistic, degrees of freedom and *P-*values for the two-way ANOVA are provided in Supplementary Table 7. Statistical differences are indicated when there is a difference from 1 month to the next *P ≤ 0.05, **P ≤ 0.01, ***P ≤ 0.001. WT = wild type, AU = arbitrary units. (**C**) Striatal sections from 6-month-old zQ175 and wild-type mice immunostained with the 2B7, S830 and MW8 antibodies. S830 detected nuclear inclusions, a diffuse nuclear aggregation signal and smaller cytoplasmic inclusions. MW8 showed a similar nuclear and cytoplasmic staining pattern to S830 but did not show the diffuse aggregation in the nucleus. 2B7 did not detect HTT aggregates. *N* = 3/genotype. Scale bar = 5 µm. WT = wild type.

To investigate whether MW8 might also be acting as a neo-epitope antibody for HTTexon1 in the aggregation assays, we again utilized the N171-82Q mouse model of Huntington’s disease that expresses a cDNA transgene containing the first 171 amino acids of *HTT*.^35^ These mice do not contain *HTT* intron 1, cannot generate *HTT1a* through the alternative processing of *HTT*, and consequently, should not produce HTTexon1. To confirm this prediction, we used cortex from zQ175 and N171-82Q mice at 2, 6 and 12 months of age. The 2B7-MW1 assay should detect all soluble mutant HTT isoforms: HTTexon1, the 171-82Q HTT protein, full-length HTT and any N-terminal proteolytic fragments. HTRF with 2B7-MW1 detected soluble mutant HTT in both models, the levels of which decreased with disease progression (Fig. 3A). In contrast, the 2B7-MW8 HTTexon1 specific assay only gave a signal in the zQ175 samples, confirming its specificity for soluble HTTexon1 (Fig. 3A).

To establish an assay that detected ‘total aggregated HTT’ (that could be comprised of any HTT proteins: full-length HTT, HTTexon1 as well as other HTT fragments extending from the N-terminus), we used the polyclonal S830 antibody as the acceptor in conjunction with the 4C9 donor. This 4C9-S830 assay detected an aggregated HTT signal that increased in both zQ175 and N171-82Q mice with disease progression (Fig. 3B). In contrast, the 4C9-MW8 and MW8-2B7 assays did not detect aggregated HTT in the N171-82Q cDNA mouse model that cannot produce HTTexon1. These assays only produced signals that tracked with HTT aggregation in zQ175 mice, consistent with their specificity for aggregated HTTexon1. Therefore, the 4C9-MW8 and MW8-2B7 signals detected from 1 to 6 months of age shown in Fig. 2 represent an increase in aggregated HTTexon1 protein.

The MW8-2B7 HTRF assay tracked HTT aggregation with lower signals than the 4C9-S830 and 4C9-MW8 assays. It only produced a signal above background in the striatum, cortex, hippocampus, cerebellum, olfactory bulb and colliculus (Fig. 2C and Supplementary Fig. 5), the brain regions for which the 4C9-S830 and 4C9-MW8 assays gave the highest signals. The MW8-2B7 assay may have a lower sensitivity than 4C9-S830 and 4C9-MW8, or alternatively, might be detecting an aggregation species that is of lower abundance. We hypothesized that the latter might be the case, as the 2B7 epitope would be expected to become buried within the aggregate assembly.^41^ To investigate this possibility, we performed immunohistochemistry on sections from 6-month-old zQ175 mice with 2B7, S830 and MW8. Aggregated HTT was readily detected by S830 and MW8, but not by 2B7 (Fig. 3C).

### The spatial appearance of aggregated HTT in zQ175 brains

We next optimized our immunohistochemical protocol for the S830 polyclonal antibody to investigate the spatial appearance of HTT aggregation throughout the zQ175 brain from 1 to 6 months of age. Sections at the level of the striatum, hippocampus and cerebellum were immunostained with S830 from 1- to 6-month-old zQ175 and wild-type mice.

**Figure 4:**
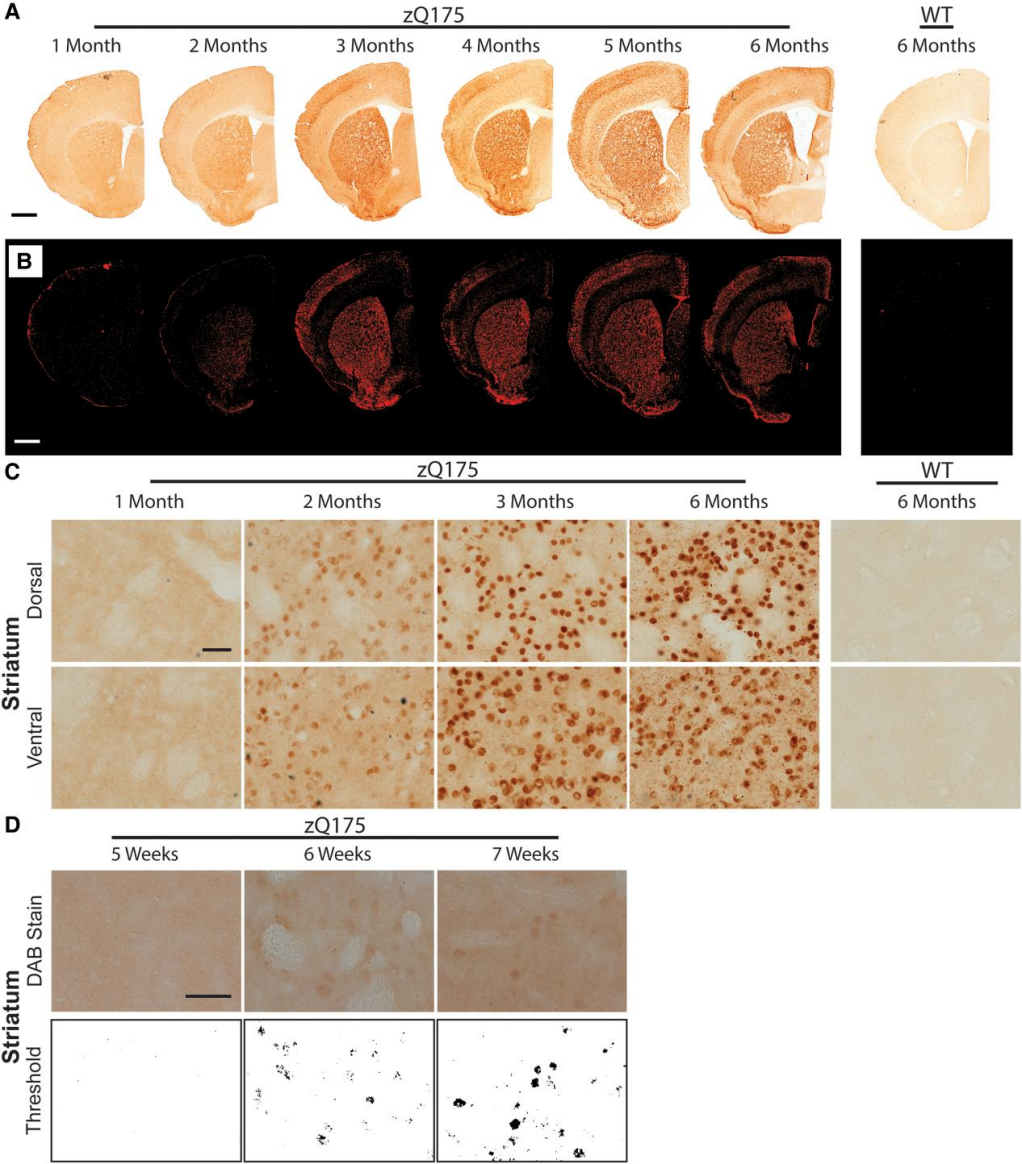
**HTT aggregation in the striatum can be detected at 6 weeks of age by immunohistochemistry.** Immunohistochemistry to coronal striatal sections from heterozygous zQ175 and wild type mice at 1–6 months of age was performed with the S830 antibody. (**A**) Low power images show the accumulation of HTT aggregation in the striatum over the 6-month time frame. Scale bar = 1 mm. (**B**) The appearance and accumulation of HTT aggregation in the striatum as observed after thresholding. Scale bar = 1 mm. **(C)** Higher power images demonstrate that aggregated HTT was clearly visible at 2 months of age and increased considerably by 3 months. A nuclear counterstain was not applied to these images, as this would mask the nuclear signal. A cresol violet counter stain, to indicate the location of the nuclei is shown in Supplementary Fig. 6. Scale bar = 20 µm. (**D**) Coronal sections from wild-type and zQ175 mice at 5, 6 and 7 weeks of age were immunostained with S830 and subjected to thresholding. HTT aggregation could be detected in the striatum at 6 weeks of age. Scale bar = 20 µm. *N* = 3. WT = wild type.

A nuclear HTT aggregation signal could be detected in the striatum at 2 months of age (Fig. 4). At low power, this could be seen to increase considerably between 2 and 3 months (Fig. 4A and B). To clearly demonstrate this, a threshold analysis was performed to distinguish the aggregation signal (red) over background (Fig. 4B). At 2 months, the signal predominantly filled nuclei, and some cytoplasmic inclusions could also be detected (Fig. 4C). By 6 months of age, the nuclear aggregate signal remained mostly diffuse with nuclear inclusions appearing in most cells, and the density of cytoplasmic inclusions had increased considerably (Fig. 4C). At 2 months, aggregation could be detected in both the ventral and dorsal striatum (Fig. 4C) and to determine when this might first become apparent, we sectioned brains from zQ175 and wild-type mice at 5, 6 and 7 weeks of age. Aggregates could be detected by 6 weeks of age in both the ventral and dorsal striatum (Fig. 4D).

**Figure 5:**
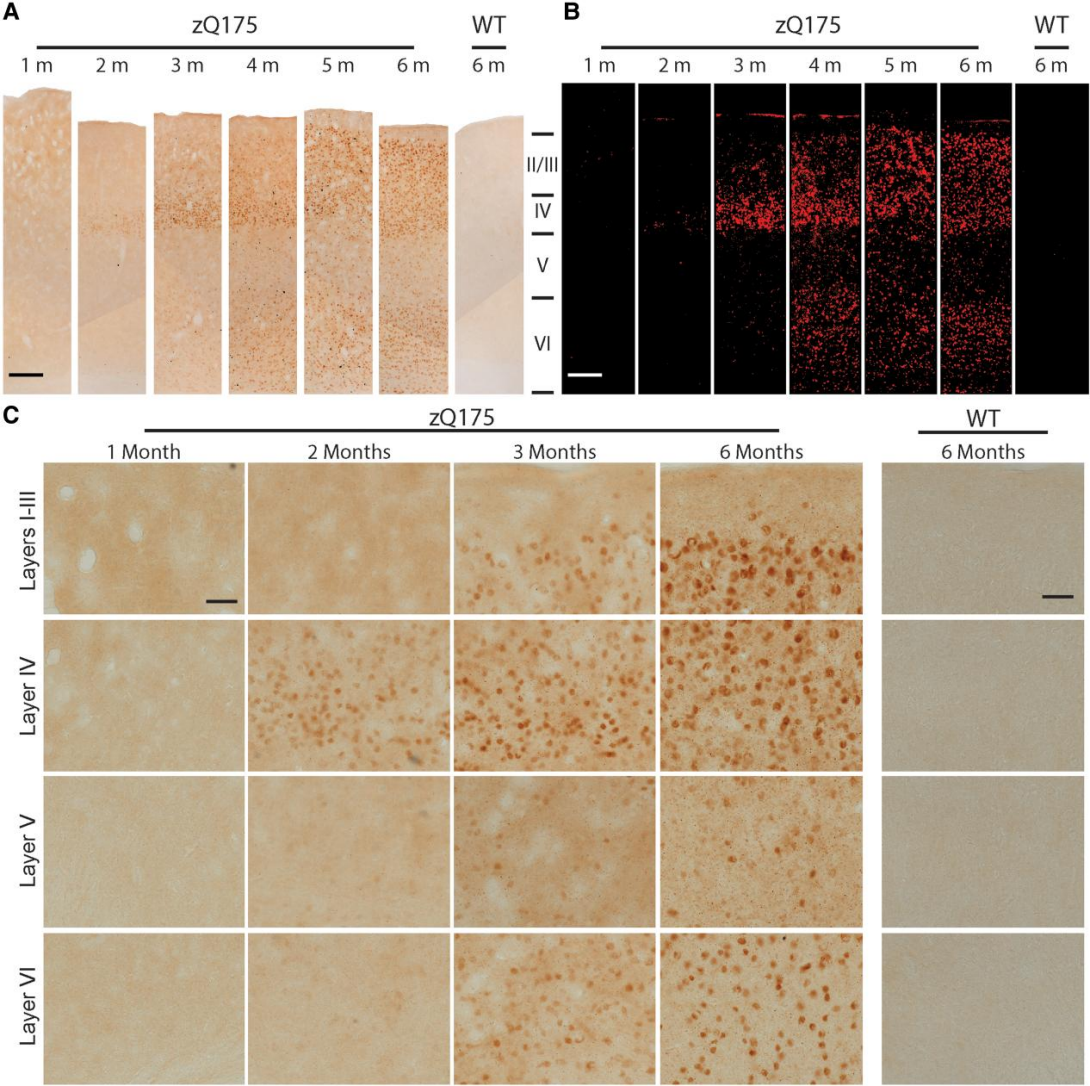
**HTT aggregates appear in layer IV of the somatosensory cortex by 2 months of age.** Immunohistochemistry to coronal sections at the level of the striatum from heterozygous zQ175 and wild-type mice at 1–6 months of age was performed with the S830 antibody. (**A**) Low power images show the accumulation of HTT aggregation in the somatosensory cortical layers over the 6-month time frame. Scale bar = 100 µm. (**B**) The appearance and accumulation of HTT aggregation throughout the layers of the somatosensory cortex after thresholding. Scale bar = 100 µm. (**C**) Higher power images demonstrate that HTT aggregates first appeared in cortical layer IV at 2 months of age and were present throughout the superficial and deep layers by 3 months. A nuclear counterstain was not applied to these images, as this would mask the nuclear signal. A cresol violet counterstain, to indicate the location of the nuclei is shown in Supplementary Fig. 6. Scale bar = 20 µm. The region of the cortex from which these images were taken is indicated in Supplementary Fig. 7. *N* = 3. WT = wild type, M = month.

Examination of the low power images at the level of the striatum (Fig. 4A and B) shows that the first appearance of HTT aggregation in the cortex occurred in one of the somatosensory cortical layers and the piriform cortex at 2 months of age. To examine the somatosensory cortex more closely, images of S830 staining, extending through the six layers, were compiled for zQ175 mice for 1–6 months of age (Fig. 5A) to which thresholding was applied (Fig. 5B). Higher power images for cortical aggregation at 1, 2, 3 and 6 months of age showed that nuclear aggregation could first be detected in layer IV at 2 months (Fig. 5C). By 3 months of age, this was also apparent in the superficial and deep cortical layers (Fig. 5C). The degree of nuclear aggregation increased from 3 to 6 months of age, remaining diffuse, with little evidence of nuclear inclusions. The density of cytoplasmic inclusions increased with age in all cortical layers, although this appeared to be less dense in layer VI (Fig. 5C).

**Figure 6:**
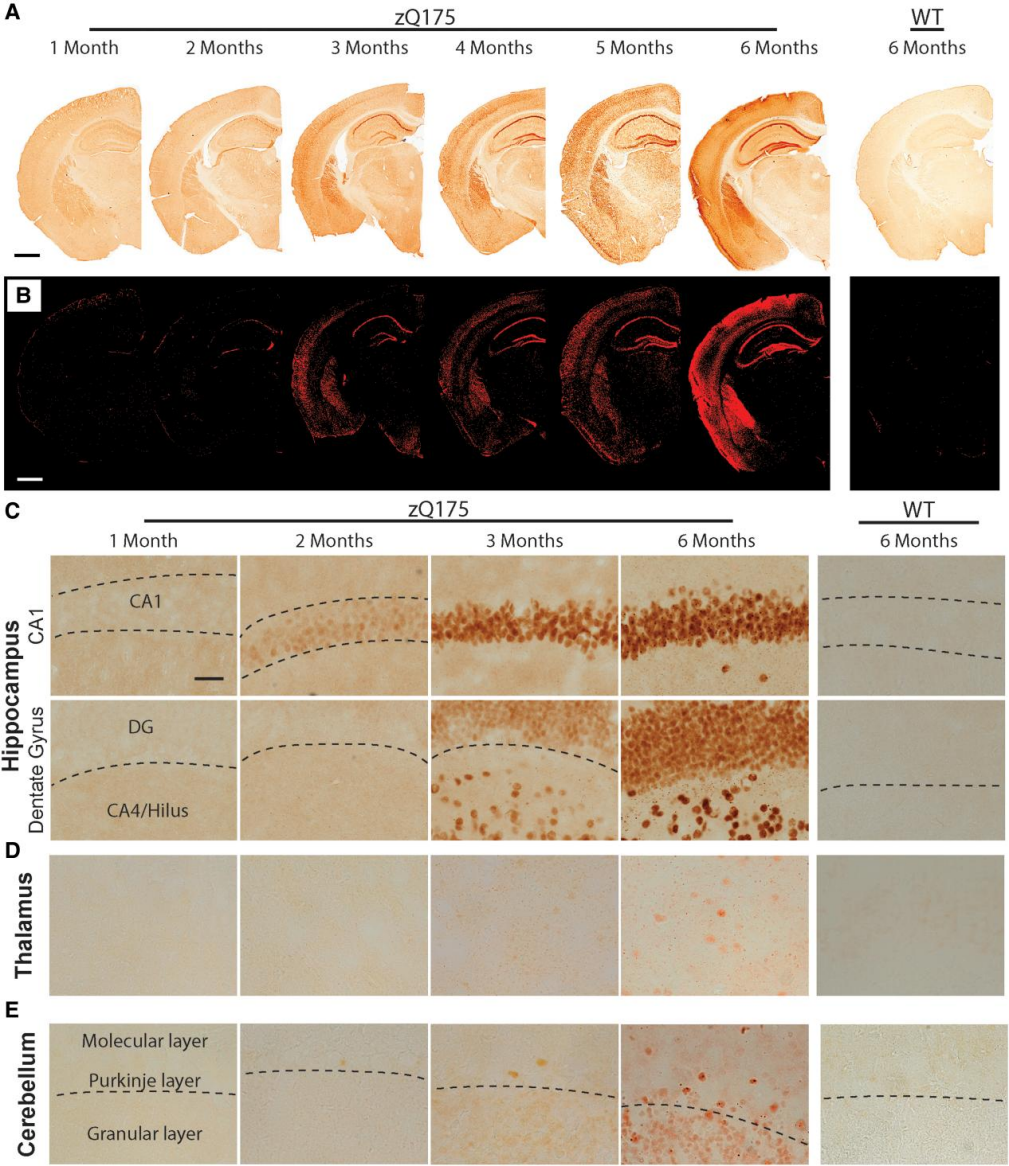
**HTT aggregation can be detected at 2 months of age in the CA1 regions of the hippocampus.** Immunohistochemistry to coronal sections at the level of the hippocampus and cerebellum from heterozygous zQ175 and wild-type mice at 1–6 months of age was performed with the S830 antibody. (**A**) Low power images show the accumulation of HTT aggregation in the hippocampus and thalamus over the 6-month time frame. Scale bar = 1 mm. (**B**) The appearance and accumulation of HTT aggregation throughout the hippocampus after thresholding. Scale bar = 1 mm. **(C)** Higher power images demonstrate that HTT aggregates first appeared in the CA1 region of the hippocampus at 2 months of age and were present in the DG and hilus by 3 months. Scale bar = 20 µm. (**D**) There was little evidence of nuclear aggregation in the thalamus and at 6 months age this remained sparse. However, cytoplasmic aggregation could be detected by 3 months of age. Scale bar = 20 µm. (**E**) In the cerebellum, HTT aggregation could be detected in the granular layer and the Purkinje cells by 3 months of age. By 6 months, nuclear inclusions were apparent in the Purkinje cells, but aggregation remained diffuse in the granular layer. Scale bar = 20 µm. A nuclear counterstain was not applied to these images, as this would mask the nuclear signal. A cresol violet counterstain, to indicate the location of the nuclei is shown in Supplementary Fig. 6. The regions of the hippocampus from which these images were taken are indicated in Supplementary Fig. 7. *N* = 3. WT = wild type, DG = dentate gyrus.

Nuclear aggregation first appeared in the pyramidal cells of the CA1 region of the hippocampus at 2 months of age (Fig. 6A). Low power images, and thresholding, show that HTT aggregation was apparent in the other pyramidal subfields and the dentate gyrus (DG) by 4 months (Fig. 6A and B). Higher power images show that at 6 months of age pyramidal cell nuclei in the CA1 regions mostly contained nuclear inclusions as well as diffuse HTT aggregation, whereas in the DG, aggregation remained diffuse (Fig. 6C). Cytoplasmic inclusions were apparent in the vicinity of the CA1 subfield and the hilus of the DG (Fig. 6C). At low power, there was little evidence of HTT aggregation in the thalamus (Fig. 6A and B), however, at higher power, although sparse nuclear aggregation was apparent, small cytoplasmic inclusions could be detected (Fig. 6D).

Within the cerebellum, diffuse HTT aggregation was present in the granular layer and Purkinje cells by 3 months of age (Fig. 6E). This had increased in intensity by 6 months and nuclear inclusions were apparent in the Purkinje cells (Fig. 6E).

### HTT aggregates were only detected in zQ175 brains with antibodies to HTTexon1 by immunostaining

We had shown in Fig. 3C that whilst both S830 and MW8 detected HTT aggregates, the 2B7 antibody, which recognized amino acids 7−13, did not. We extended this analysis to another antibody that detected the first 17 amino acids of HTT (EPR5526) as well as antibodies that recognized epitopes located C-terminal to HTTexon1 in the full-length HTT protein: MAB5490, MAB2166 and D7F7 (Fig. 1). None of these antibodies detected aggregated HTT in sections from 6-month-old zQ175 mice; in all cases, the background was comparable to that observed on wild-type sections (Supplementary Fig. 8).

### The diffuse nuclear immunostain detected by S830 represents an aggregated form of HTT in zQ175 brains

Our immunohistochemical analyses with S830 most frequently detected a diffuse staining pattern in cell nuclei (Figs. 4–6), which was never detected in wild-type sections. In the striatum and CA1 regions of the hippocampus, nuclear inclusions could be detected within this diffuse HTT immunostain at 6 months of age, but in the cortex and DG, there was little evidence of nuclear inclusions, even at 6 months.

**Figure 7:**
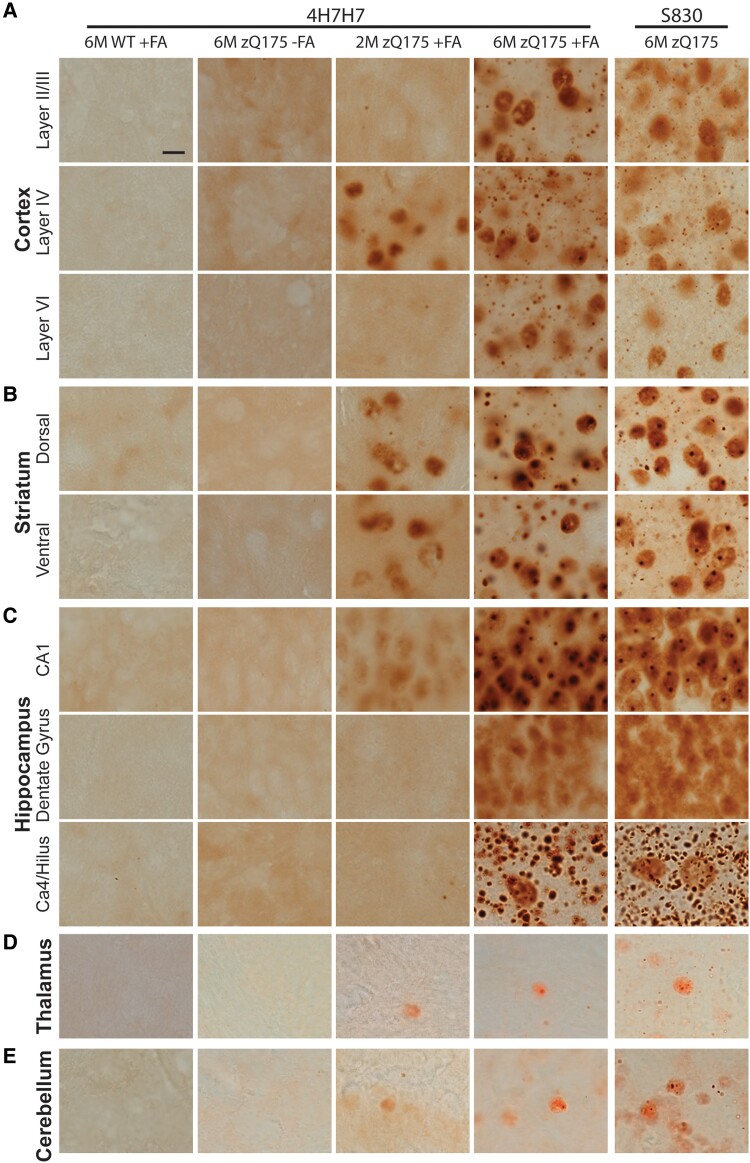
**Confirmation of HTT aggregate staining with formic acid antigen retrieval.** (**A–E**) Immunohistochemistry with the 4H7H7 antibody to sections from heterozygous zQ175 mice at 6 months did not detect HTT aggregation (column 2) in any brain region. 4H7H7 immunostaining after pretreatment with formic acid revealed HTT aggregation in 2-month and 6-month-old zQ175 sections (columns 3 and 4), but not in sections from 6-month-old wild-type mice (column 1). S830 immunostaining of sections from 6-month-old zQ175 mice is shown for comparison (column 5). Consistent with S830 staining, immunoprobing with 4H7H7 after formic acid antigen retrieval only detected HTT aggregation at 2 months of age in (**A**) layer IV of the somatosensory cortex and not the superficial or deep layers, (**B**) the striatum and (**C**) the CA1 region of the hippocampus and not the DG or hilus. (**A–C**) In general, the immunostaining of zQ175 sections at 6 months of age with 4H7H7 post formic acid treatment was more intense than with S830. In the cortical layer, nuclear inclusions were more apparent with 4H7H7, and in all regions, larger and easier to detect. However, overall, treatment with formic acid did not reveal a pattern of aggregation that had not been detected with S830. (**D, E**) Pretreatment with formic acid and 4H7H7 immunolabelling did not enhance the extent to which HTT aggregation had been detected with S830 in the thalamus or cerebellum. *N* = 3. Scale bar 5 µm. WT = wild type, M = months, FA = formic acid.

To investigate whether this diffuse nuclear immunostain represented an aggregated form of HTT, we employed formic acid antigen retrieval.^42,43^ The 4H7H7 antibody detects polyglutamine and, when applied to 6-month-old zQ175 sections, no HTT-specific signal was detected in the cortex (Fig. 7A), striatum (Fig. 7B), hippocampus (Fig. 7C) thalamus (Fig. 7D) or cerebellum (Fig. 7E). However, if the sections were pretreated with formic acid to disrupt the amyloid structure,^44^ the staining pattern observed with S830 was revealed (Fig. 7). There was no 4H7H7 immunostain on wild-type sections that had been pretreated with formic acid (Fig. 7). Therefore, the diffuse nuclear HTT immunostain detected with S830 represents an aggregated form of HTT.

The S830 immunostained sections were compared to those stained with 4H7H7 after polyQ antigen retrieval. At 6 months of age, the diffuse nuclear 4H7H7 staining was more pronounced than that with S830, and the polyQ antigen retrieval revealed a high density of nuclear inclusions in cortical layer IV that were not previously apparent. The presence of cytoplasmic inclusions was more prominent in all brain regions after polyQ antigen retrieval, especially in the hilus, where they were shown to be densely packed. However, despite this apparent increased sensitivity, the first appearance of HTT aggregation, as detected by 4H7H7 after polyQ antigen retrieval was comparable to that detected by S830: the striatum, layer IV for the cortex and the CA1 region of the hippocampus (Figs. 4C, 5C, 6C and 7).

The epitope recognized by 2B7 (7–13 amino acids) is adjacent to the polyQ tract. Therefore, we repeated the formic acid antigen retrieval to investigate whether this approach might expose the 2B7 epitope as well as polyglutamine. Sections from zQ175 and wild-type mice were treated with formic acid and immunoprobed with 4H7H7 and with 2B7. This treatment exposed the epitope for 4H7H7 but not for 2B7 (Supplementary Fig. 9).

### Soluble HTTexon1 levels decrease in zQ175 brains with disease progression between 1 and 6 months of age

**Figure 8:**
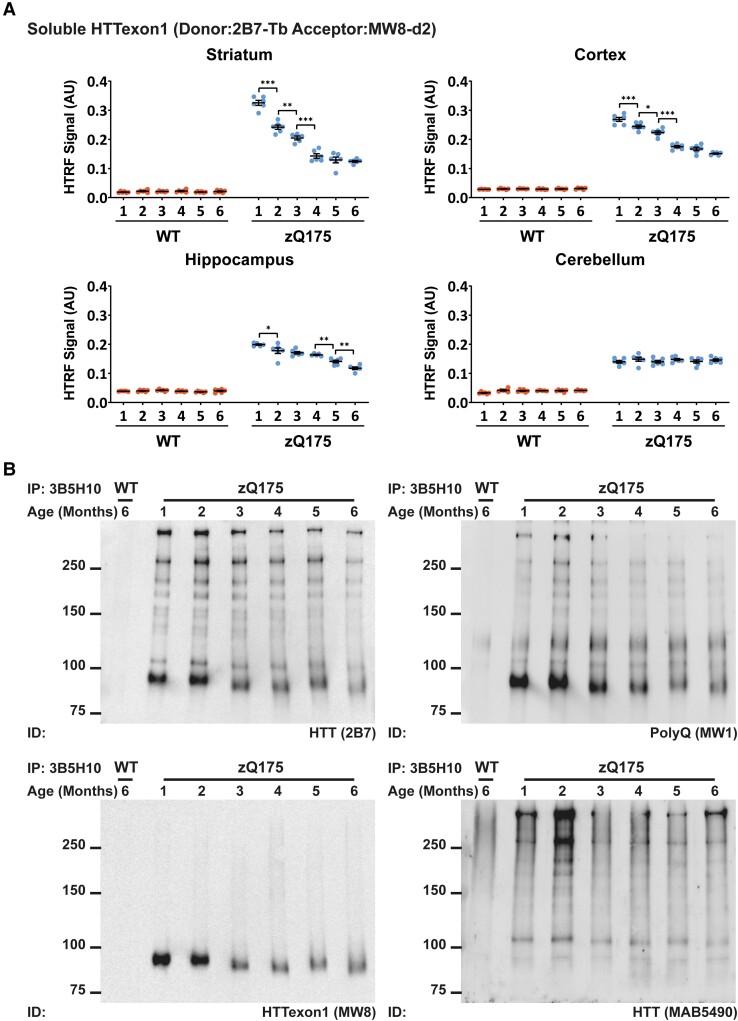
**The level of soluble HTTexon1 decreases with disease progression in zQ175 CNS regions.** (**A**) The level of soluble HTTexon1, as detected by the 2B7-MW8 HTRF assay decreased in all heterozygous zQ175 brain regions from 1 to 6 months of age except for cerebellum. The complete HTRF assay data set for all nine CNS regions is shown in Supplementary Fig. 10. The brain regions with the highest levels of HTTexon1 were broadly comparable to those with the highest levels of HTT aggregation, as measured by HTRF. *N* = 6/genotype/age. Error bars are mean ± SEM. The test statistic, degrees of freedom and *P-*values for the two-way ANOVA are provided in Supplementary Table 8. Statistical differences indicate when there is a difference from 1 month to the next and to indicate the age at which the level of soluble HTTexon1 were first found to decrease *P ≤ 0.05, **P ≤ 0.01, ***P ≤ 0.001. WT = wild type, AU = arbitrary units. (**B**) Mutant HTT was immunoprecipitated from cortical lysates with the 3B5H10 antibody that detects expanded polyQ. Western blots were immunoprobed with 2B7 (amin acids 7–13), MW1 (polyQ), MW8 (HTTexon1) and MAB5490 (amino acids 115–129). The level of HTTexon1 decreased in cortical lysates from zQ175 mice over the 6-month time frame as compared to full-length HTT and its proteolytic fragments. Please note that the samples from 2-month-old mice are relatively over-loaded. WT = wild type. IP = immunoprecipitated. ID = immunodetect.

In zQ175 mice, *HTT* mRNA is processed to generate the full-length *Htt* transcript as well as *Htt1a* that encodes the HTTexon1 protein. We applied the 2B7-MW8 HTRF assay to determine whether the levels of soluble HTTexon1 change with disease progression in nine CNS regions from zQ175 mice from 1 to 6 months of age (Fig. 8A and Supplementary Fig. 10). It was not possible to include the hypothalamus as this had all been used for the aggregation assays. Soluble HTTexon1 was present in all nine CNS regions and decreased over the 6-month period in all cases, except for cerebellum for which the levels remained constant. In general, the brain regions with the highest levels of HTTexon1 correlated with those with the highest levels of aggregation as detected by HTRF (Supplementary Figs. 3, 4, 5 and 10).

There was sufficient cortical tissue to perform an immunoprecipitation to visualize the decrease in HTTexon1. Mutant HTT was immunoprecipitated from cortical lysates with 3B5H10 (that binds to expanded polyQ tracts), fractionated on 8% SDS-polyacrylamide acrylamide gel electrophoresis gels, western blotted and immunoprobed with 2B7, MW1, MW8 and MAB5490 (Fig. 8B). 2B7 binds to an epitope within the first 17 amino acids of HTT and therefore, detects full-length HTT and all N-terminal HTT fragments (Fig. 8B). MW1 binds to polyQ, and similarly detects full-length HTT and all N-terminal fragments. The MW1 bands in the wild-type lane most likely represent other polyQ-containing proteins, as antibodies to the polyQ tract had been used for both immunoprecipitation and immunodetection. MW8 is a neo-epitope antibody for HTTexon1,^39^ confirming that the single band on the MW8 blot represented HTTexon1. MAB5490 detects an epitope that is C-terminal to HTTexon1 and therefore did not identify the HTTexon1 protein (Fig. 8B). The MW8 blot confirmed that the level of HTTexon1 decreased with disease progression in the zQ175 cortex.

### Full-length mutant HTT levels remain unchanged in nine CNS regions from zQ175 mice from 1 to 6 months of age

**Figure 9:**
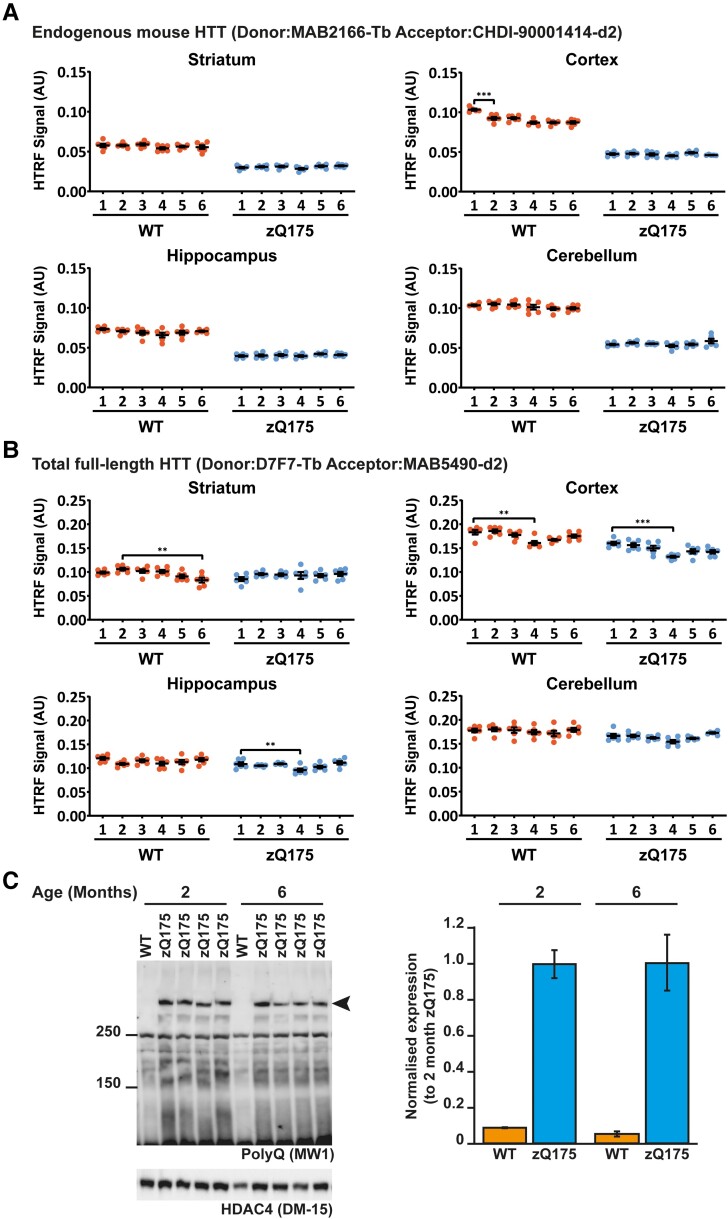
**The level of full-length HTT does not change in zQ175 brains from 1 to 6 months of age.** (**A**) The level of endogenous mouse HTT, as detected by the MAB2166-CHDI-1414, was higher in wild-type mice that have two copies of mouse HTT compared to heterozygous zQ175 mice that have one copy. The complete HTRF assay data set for all nine CNS regions is shown in Supplementary Fig. 1^1^. There was no change in the levels of endogenous mouse HTT in zQ175 mice. *N* = 6/genotype/age. Error bars are mean ± SEM. The test statistic, degrees of freedom and *P-*values for the two-way ANOVA are provided in Supplementary Table 9. The one statistical difference is shown ***P ≤ 0.001. WT = wild type, AU = arbitrary units. (**B**) Total full-length HTT (mutant and wild-type) was detected using the D7F7-MAB5490 assay. The complete HTRF assay data set for all nine CNS regions is shown in Supplementary Fig. 1^2^. The levels of total full-length HTT were comparable between wild-type and zQ175 mice with no evidence that the mutation resulted in a reduction in full-length HTT levels. *N* = 6/genotype/age. Error bars are mean ± SEM. The test statistic, degrees of freedom and *P-*values for the two-way ANOVA are provided in Supplementary Table 10. Statistical differences indicate when there was a difference from 1 month to the next, and the age at which changes were first detected **P ≤ 0.01, ***P ≤ 0.001. WT = wild type, AU = arbitrary units. (**C**) Western blot of cortical lysates from zQ175 mice at 2 and 6 months of age probed with MW1 to detect mutant full-length HTT. HDAC4 was used as a loading control (see Supplementary Fig. 1^3^ for full-length blot). Size standards are in kDa. Quantification howed that the levels of mutant HTT were comparable between 2- and 6-month-old zQ175 cortices. *N* = 4. WT = wild type.

To investigate whether the levels of soluble full-length mutant HTT change in nine CNS regions from zQ175 mice from 1 to 6 months of age, we employed two HTRF assays: MAB2166-CHDI-1414 that detects endogenous mouse HTT and D7F7-MAB5490 that detects total HTT levels (mutant and wild-type) (Fig. 9A and B and Supplementary Figs. 11 and 12). The levels of endogenous mouse HTT remained unchanged from 1 to 6 months in all nine CNS regions from zQ175 mice that we had analysed (Fig. 9A and Supplementary Fig. 1^1^). Changes in total soluble HTT levels occurred in four CNS regions for wild-type mice and five regions for zQ175 mice. In the cortex, colliculus and spinal cord, a comparable decrease in total HTT levels occurred in wild-type and zQ175 mice (Supplementary Fig. 1^2^). Therefore, this decrease cannot be caused by the Huntington’s disease mutation and is likely to be a reflection of technical variables.

To confirm that full-length mutant HTT levels had not changed in the zQ175 cortex, we performed western blotting on 2- and 6-month lysates and immunoprobed with MW1. Mutant HTT levels had not changed in the cortex of zQ175 mice between 2 and 6 months of age (Fig. 9C).

## Discussion

In the context of an expanded CAG repeat, two transcripts are produced from the *HTT* pre-mRNA: full-length *HTT* and *HTT1a* that is generated by polyadenylation and termination at cryptic polyA sites within intron 1. *HTT1a* encodes the aggregation-prone HTTexon1 protein that is known to be highly pathogenic.^21,45^ HTT-lowering strategies that are currently being evaluated in clinical trials target either just full-length *HTT* or both full-length *HTT* and *HTT1a*.^22^ It is essential that the consequences of these interventions on the levels of soluble and aggregated HTT isoforms can be assessed in preclinical analyses. In this study, we have developed three HTRF assays to detect HTT aggregation, two of which are specific for HTTexon1. Given zQ175 knock-in mice are a model of choice for preclinical studies, we have used our HTRF assays for soluble and aggregated HTT isoforms together with immunohistochemistry to map the formation and accumulation of HTT aggregation throughout the zQ175 mouse brain from 1 to 6 months of age. We found that HTT aggregation can be detected at between 1 and 2 months of age in all 10 CNS regions assessed. This detailed analysis provides essential baseline information for future HTT-lowering preclinical trials.

The zQ175 mouse model of Huntington’s disease is used for preclinical studies because of its comparatively early phenotype onset compared to other knock-in mouse models.^30,31,46^ Even so, there is little evidence of progressive motor or cognitive impairments in heterozygous zQ175 mice before 10–12 months of age.^30,31^ Although a decrease in MRI striatal and cortical volume has been reported from 4 months,^31^ this did not correlate with cell death, as there was no change in neuronal number, neuronal density, cross-sectional area or regional volume in the striatum, up to 10 months of age.^31^ The earliest phenotypes to be reported included changes in some electrophysiological parameters by 3–4 months of age^31^ and transcriptional dysregulation in many brain regions at 6 months, but not at 2 months of age.^47^ We have shown that HTT aggregation preceded these phenotypes and could be readily detected at 1 to 2 months of age in all 10 CNS regions assessed by utilizing HTRF assays.

We used immunohistochemistry with the S830 antibody to map the regional and subcellular location and accumulation of HTT aggregation. In the cortex, striatum and hippocampus, HTT aggregation was visualized by 2 months of age, comparable to the age at which aggregation was detected by HTRF. This is slightly earlier than reported for the quantification of HTT aggregation in zQ175 brains using an automated analysis of immunostained sections with fluorescently labelled EM48.^48^ In all cases, the entire nucleus was filled by a diffuse S830 immunostain that was apparent up to 6 months of age, during which time, nuclear inclusions formed in many, but not all, nuclei. We were able to demonstrate the diffuse immunostain represented aggregated HTT by probing with 4H7H7 after formic acid retrieval of the polyQ epitope. We have previously shown that in R6/2 mice with 90 CAGs and YAC128 mice, HTT aggregation in the nucleus remained relatively diffuse throughout disease progression.^36,49^ We have also previously demonstrated that, in HTTexon1 and knock-in mouse models of Huntington’s disease, the steady-state levels of soluble HTT isoforms are cytoplasmic and that HTT fragments remain in the nucleus because they have aggregated into high-molecular-weight complexes.^36,39,50^ Here, we have shown that HTT aggregation in zQ175 striatal nuclei was present as early as 6 weeks of age, well before transcriptional dysregulation has been reported^47^ and consistent with transcriptional dysregulation being caused by aggregated HTT.^36^ Cytoplasmic aggregation was found throughout the zQ175 brain but was particularly prominent in some regions such as the hilus of the hippocampus. In the thalamus, nuclear aggregation was sparse, and although cytoplasmic aggregation may have been present in cell bodies, it may have been deposited in the neuronal networks projecting to this region e.g. from layer VI of the cortex.

The data presented here provide further evidence to indicate that MW8 is a conformation-specific antibody which, under many experimental conditions, acts as a neo-epitope antibody for HTTexon1.^34,39,51^ We had previously demonstrated that the successful recognition of HTT on western blots by MW8 depended on the presence of a free proline residue at the C-terminus of the HTTexon1 protein.^39^ We also found that the 2B7-MW8 HTRF, MSD and AlphaLISA assays were specific to soluble HTTexon1.^34^ Here we demonstrate that the 4C9-MW8 and MW8-2B7 HTRF assays are specific for aggregated HTTexon1, which we would predict would also be the case for the previously established MW8-4C9 MSD assay.^52^

The HTTexon1 specific MW8-2B7 aggregation assay may be less sensitive than the 4C9-S830 and 4C9-MW8 assays, as it only detected HTT aggregation in brain regions in which these two assays indicated that the most aggregation had occurred. Alternatively, it could be measuring a different HTT aggregate species. The 2B7 antibody binds to the first 17 amino acids of HTT and we were not able to detect aggregated HTT by immunohistochemistry with either 2B7 (Fig. 3C) or with EPR5526 (Supplementary Fig. 8) consistent with a model in which N17 becomes buried during the aggregate assembly.^41^ Therefore, the MW8-2B7 assay may be detecting an HTT oligomer, via an intramolecular fluorescence resonance energy transfer (FRET) event, that is produced early in the aggregation cascade. Alternatively, the aggregation HTRF assays may be detecting FRET events resulting from intermolecular interactions, in which case, the MW8-2B7 assay may reflect polymerization events in which HTT monomers (2B7) are recruited to HTT aggregates (MW8). The latter case would represent a seeding event. However, our measurement of HTT seeding using the FRASE assay^38^ gave a different profile across brain regions to the HTRF assays, possibly arguing against this.

Our data are consistent with the hypothesis that it is the HTTexon1 protein that initiates HTT aggregation in zQ175 mice. Overall, the level of soluble HTTexon1 decreased with disease progression whereas full-length HTT levels remained constant, suggesting that HTTexon1 was being recruited into aggregates. Our 4C9-MW8 HTRF assay indicated that HTT aggregation in all zQ175 brain regions contained HTTexon1 and that the HTTexon1 fragment would aggregate readily is consistent with *in vitro*^20,53^ and *in vivo*^38,54^ data. The only brain region for which soluble HTTexon1 levels remained constant was the cerebellum; the region that gave the highest FRASE signal, and for which immunohistochemistry detected diffuse aggregation in the nuclei of granule and Purkinje cells. The FRASE signal may reflect the high density of granule cells in this brain region, and consequently, a greater number of aggregate seeds. If these polymerized by recruiting HTTexon1 at a lower rate than in other brain regions, a progressive reduction in soluble HTTexon1 might not be detected.

It might be expected that other HTT fragments, generated by proteolytic cleavage or the full-length protein, would also be recruited into these structures. Consistent with previous data, we were unable to detect HTT aggregates by immunohistochemistry with antibodies that are C-terminal to HTTexon1.^55^ This absence of detection may be due to epitope masking, or because HTT fragments longer than HTTexon1 are present at levels too low to be detected by immunohistochemistry. In support of the latter, we previously detected the accumulation of low levels of HTT fragments longer than HTTexon1 in zQ175 brains, consistent with their presence in aggregates, with the MSD assays MW8-MAB5490 and MW8-MAB2166, but not with the corresponding HTRF antibody pairings.^34^

We have used molecular and immunohistochemical techniques to conduct a detailed analysis of HTT aggregation in the brains of zQ175 mice up to 6 months of age, well before the advent of behavioural phenotypes. Aggregation could be detected in very young mice: at 1 month of age in all brain regions by HTRF and could be visualized in the striatum and regions of the cortex and hippocampus by 2 months. Aggregation in the zQ175 brain is complex. In the nucleus, it first appears as a diffuse aggregation that may coalesce to form nuclear inclusions and the relative levels of nuclear and cytoplasmic aggregates differ between brain regions. It has already been shown that treatment of zQ175 mice with a zinc-finger protein targeting mutant *HTT* expression had a more pronounced effect on HTT aggregation when administered at 2 months of age as opposed to 6 months, the reduction in nuclear aggregation being greatly diminished after treatment at 6 months.^26^ Therefore, it is essential that the levels of soluble and aggregated HTT isoforms are tracked in response to HTT-lowering interventions, irrespective of the target. The HTRF assays provide a rapid means of measuring bulk levels of soluble HTTexon1 and full-length HTT as well as total HTT aggregation and aggregated HTTexon1 in tissue lysates. However, these must be complemented with an immunohistochemical approach to visualize nuclear and cytoplasmic aggregation patterns, to allow an understanding of the impact of HTT-lowering strategies dependent on their target and the stage of disease at which they are administered.

## Supplementary Material

fcad010_Supplementary_Data

## Data Availability

The authors confirm that all the data supporting the findings of this study are available within the article and its Supplementary material. Raw data will be shared by the corresponding author upon request.

## Abbreviations

CAG =: cytosine, adenine, guanine
DAB =: 3.3′-diaminobenzidine
ΔF =: change in fluorescence
FRASE =: FRET-based aggregate seeding assay
FRET =: fluorescence resonance energy transfer
HTRF =: homogeneous time-resolved fluorescence
HTTexon1 =: exon 1 HTT protein
MSD =: meso scale discovery
PBS =: phosphate buffer saline
PolyA =: polyadenylation
PolyQ =: polyglutamine
Q =: glutamine
SEM =: standard error of the mean
