# Supplementary material for: Early detection of exon 1 huntingtin aggregation in zQ175 brains by molecular and histological approaches
Source: Brain Commun. 2023 Jan 20;5(1):fcad010. doi: 10.1093/braincomms/fcad010 (PMC9901570; doi:10.1093/braincomms/fcad010)
Supplement: fcad010_Supplementary_Data [file fcad010_supplementary_data.pdf]

SUPPLEMENTARY MATERIAL

Early detection of exon 1 huntingtin aggregation in zQ175 brains by molecular and histological approaches

Supplementary Table 1. Summary of the antibodies used

Name	Immunogen	Epitope	Species	Dilution IHC	Source / reference
2B7	HTT peptide: amino acids 1-17	aa 7-13	Mouse monoclonal	1:500	CHDI Foundation[1]
EPR5526	N-terminal HTT peptide	Within aa 1-17	Rabbit monoclonal	1:500	Abcam ab109115
MW1	Exon 1 HTT (67Q)	PolyQ	Mouse monoclonal	1:500	CHDI Foundation[2]
3B5H10	PolyQ	PolyQ	Mouse monoclonal	N/A	Sigma-Aldrich P1874
Biotinylated 4H7H7	PolyQ	PolyQ	Mouse monoclonal	1:3000	Ron Wetzel
4C9	HTT peptide: amino acids 51-71		Mouse monoclonal	1:500	CHDI Foundation[3]
CHDI-90001414	Mouse proline-rich region of HTTexon1		Rabbit polyclonal	N/A	CHDI Foundation
S830	Exon 1 HTT (53Q)		Sheep polyclonal	1:2000	In-house[4]
MW8	Exon 1 HTT (67Q)	aa 83-90	Mouse monoclonal	1:500	CHDI Foundation[2]
MAB5490	Recombinant HTT: amino acids 115-129		Mouse monoclonal	1:500	Sigma-Aldrich, MAB5490
MAB2166	Recombinant HTT: amino acids 181-810	aa 443-457[5]	Mouse monoclonal	1:500	Sigma-Aldrich, MAB2166
D7F7	HTT peptide:	Residues surrounding Pro1220	Rabbit monoclonal	1:500	Cell Signalling Technology mAb#5656
HDAC4 (DM-15)			Rabbit polyclonal	N/A	Merck H9536

IHC = immunohistochemistry; aa = amino acid; Pro = proline

Supplementary Table 2. Optimized conditions for the HTRF assays.

zQ175 Assay Type	Antibody pairing (Donor : Acceptor)	Acceptor antibody	Lysate dilution
Total HTT aggregation	4C9-Tb : S830-d2	40 ng	10%
HTTExon1 aggregation	4C9-Tb : MW8-d2	10 ng	10%
HTTExon1 aggregation	MW8-Tb : 2B7-d2	40 ng	10%
Soluble HTTexon1	2B7-Tb : MW8-d2	40 ng	10%
Total full-length HTT	D7F7-Tb : MAB5490-d2	20 ng	5%
Endogenous mouse HTT	MAB2166-Tb : CHDI-90001414-d2	40 ng	10%

Tb = terbium

Supplementary Figure 1

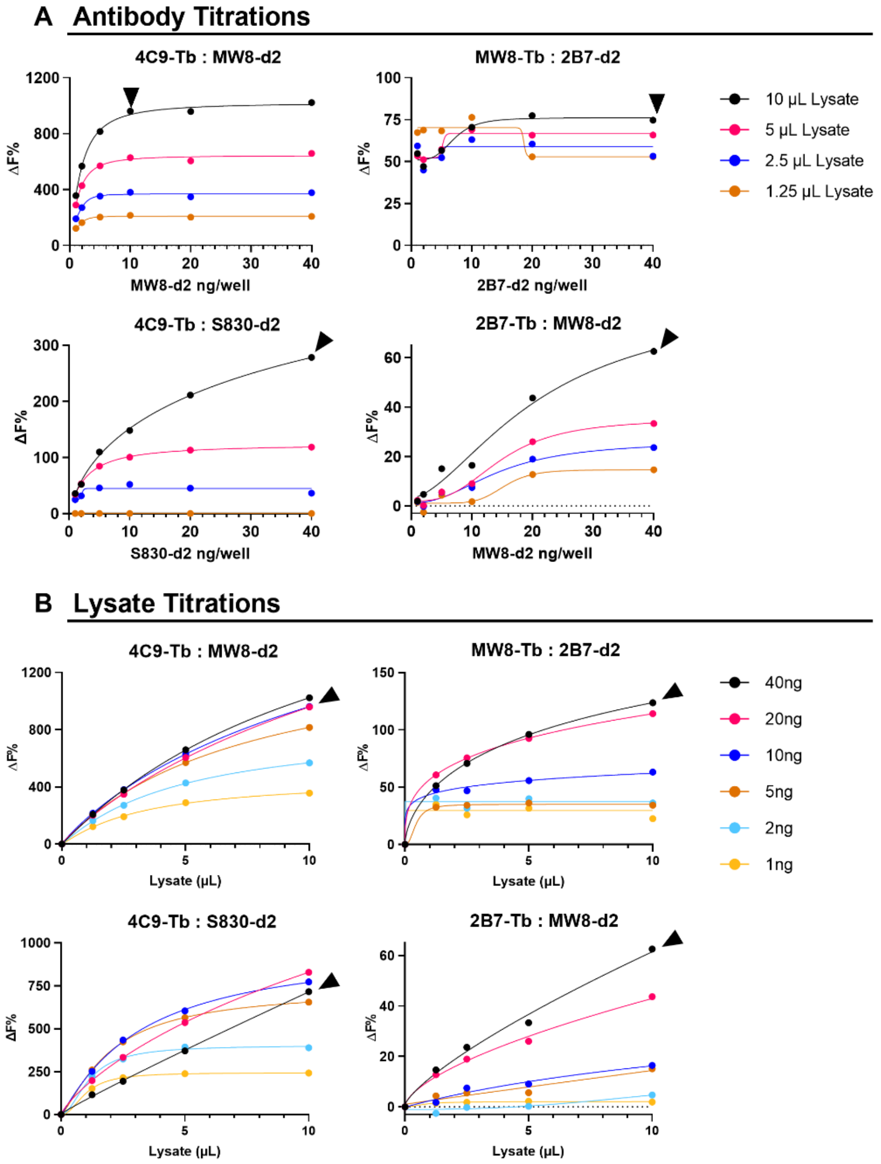

Supplementary Figure 1. Optimization of antibody concentrations and lysate titrations for use in the three HTRF aggregation assays and the soluble HTTexon1 protein assay in zQ175 tissues. (A) Optimization of antibody concentrations was performed on cortical lysates from 12-month-old zQ175 mice for assays that detect aggregated HTT: 4C9-S830, 4C9-MW8 and MW8-2B7 and from 2-month-old zQ175 mice for the soluble HTTexon1 assay. The donor antibody concentration was kept constant at 1 ng/well, whilst the acceptor antibody concentration was titrated from 1 ng/well to 40 ng/well. The maximum concentration prior to saturation was chosen as optimal (arrow). **(B)** Two-fold serial dilutions of 1.25 – 10 µL cortical lysate from zQ175 mice was performed by diluting with age-matched wild-type lysate. The titration curve for the optimal antibody concentration is indicated (arrow). The change in fluorescent signal is denoted as ΔF%. N = 3.

Supplementary Figure 2

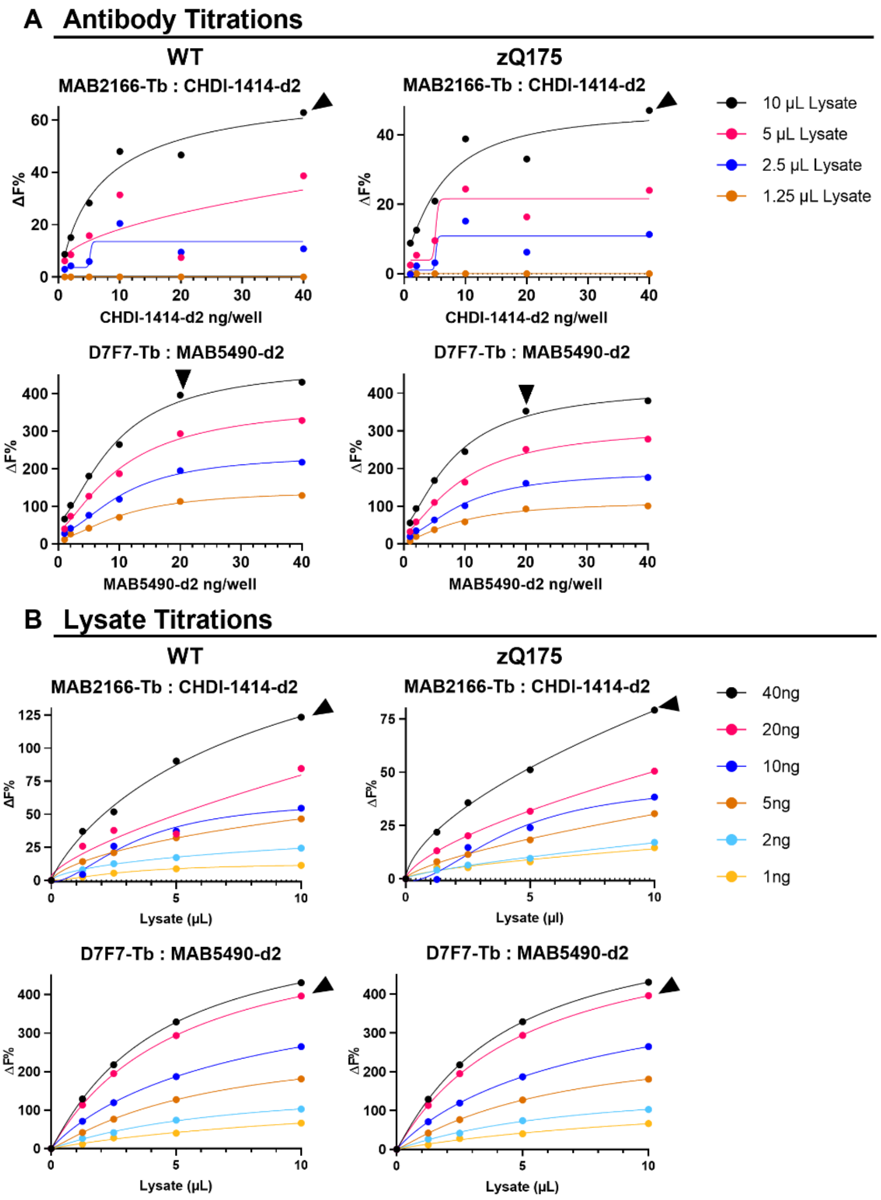

Supplementary Figure 2. Optimization of antibody concentrations and lysate titrations for use in the 'endogenous mouse HTT' and 'total full-length HTT' HTRF assays. (A) Optimization of antibody concentrations was performed on cortical lysates from 6-month-old wild-type and zQ175 mice for the assays that detect soluble wild-type endogenous mouse HTT: MAB2166-CHDI-1414 and total soluble full-length HTT (mutant and wild-type): D7F7-MAB5490. The donor antibody concentration was kept constant at 1 ng/well, whilst the acceptor antibody concentration was titrated from 1 ng/well to 40 ng/well. The maximum concentration prior to saturation was chosen as optimal (arrow). **(B)** Two-fold serial dilutions of 1.25 – 10 µL cortical lysate from zQ175 mice was performed by diluting with lysis buffer. The titration curve for the optimal antibody concentration is indicated (arrow). The change in fluorescent signal is denoted as ΔF%. N = 3. WT = wild type.

Supplementary Figure 3

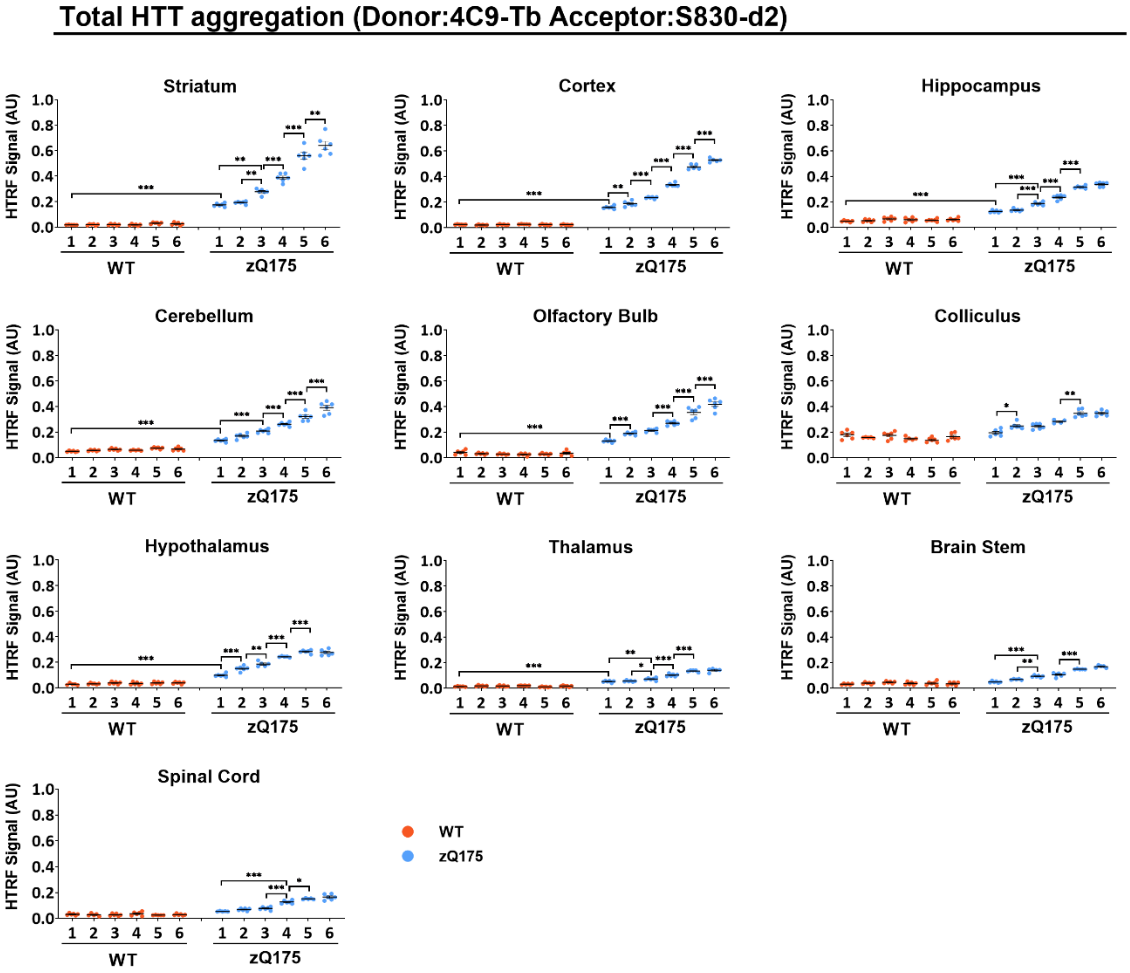

Supplementary Figure 3. Comparative increase in aggregated HTT in ten CNS regions from zQ175 mice aged from 1 – 6 months. Aggregated HTT, as detected by the 4C9-S830 assay, increased in all brain regions from 1 – 6 months of age. The presence of aggregated HTT could be first detected at 1 month in all regions except for colliculus, brain stem and spinal cord. The greatest level of aggregated HTT was in the striatum followed by the cortex, with the lowest levels in the thalamus and brain stem. In four of the brain regions, aggregated HTT levels continued to increase up to 6 months of age, whereas in the others, the levels had started to plateau between 5 and 6 months. N = 6 / genotype / age. Error bars are mean ± SEM. The test statistic, degrees of freedom and *p* values for the two-way ANOVA are provided in Supplementary Table 3. Statistical differences are indicated when there is a difference from one month to the next and to indicate the age at which aggregated HTT could first be detected *$p \leq 0.05$, **$p \leq 0.01$, ***$p \leq 0.001$. WT = wild type, AU = arbitrary units.

Supplementary Figure 4

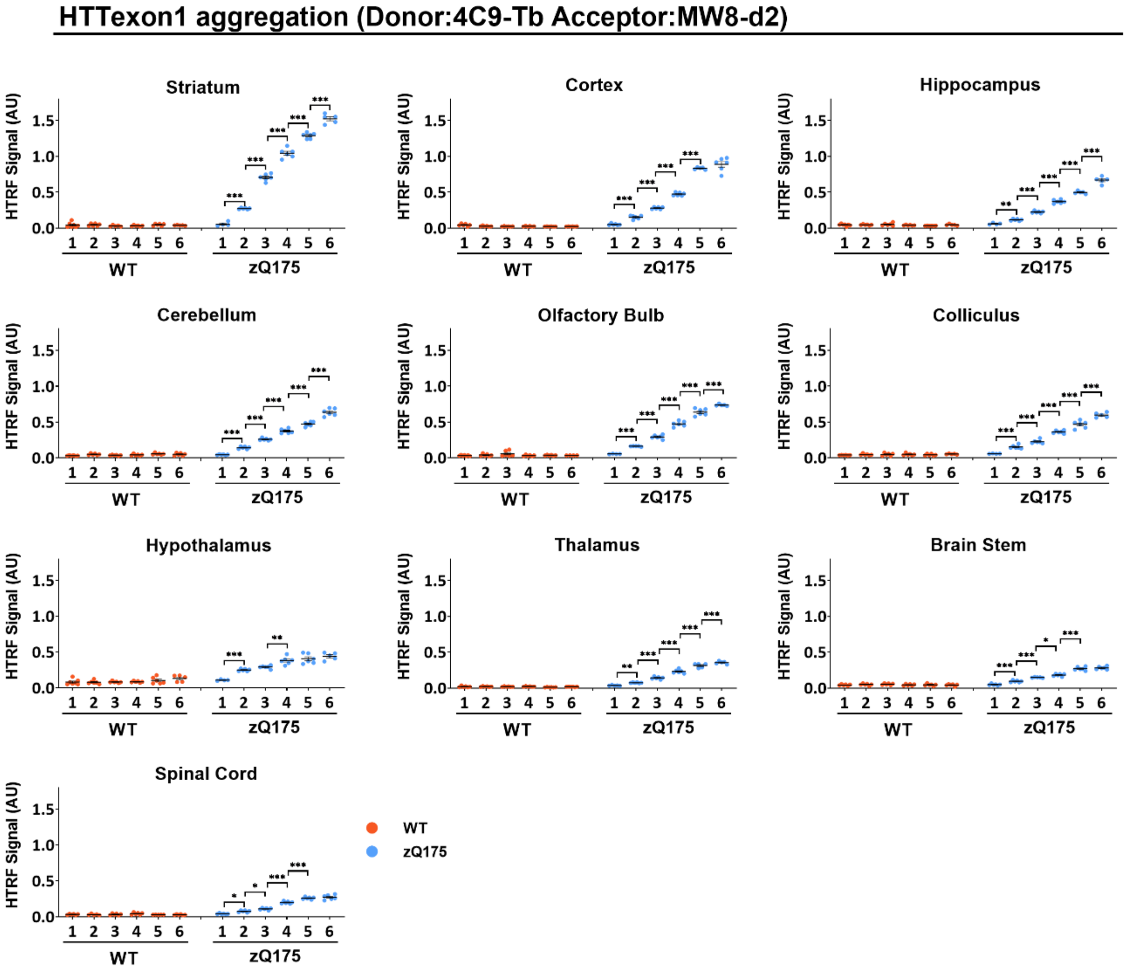

Supplementary Figure 4. Comparative increase in aggregated HTTexon1 in ten CNS regions from zQ175 mice aged from 1 – 6 months. Aggregated HTTexon1, as detected by the 4C9-MW8 assay increased in all brain regions from 1 – 6 months of age. In all cases, the presence of aggregated HTTexon1 could be first detected above background at 2 months. The greatest level of aggregated HTTexon1 was in the striatum followed by the cortex, with the lowest in the brain stem and spinal cord. In seven of the brain regions, the levels had started to plateau between 5 and 6 months, although aggregated HTT continued to increase up to 6 months of age in the hippocampus, cerebellum and thalamus. N = 6 / genotype / age. Error bars are mean ± SEM. The test statistic, degrees of freedom and *p* values for the two-way ANOVA are provided in Supplementary Table 4. Statistical differences are indicated when there is a difference from one month to the next and to indicate the age at which aggregated HTTexon1 could first be detected *$p \leq 0.05$, **$p \leq 0.01$, ***$p \leq 0.001$. WT = wild type, AU = arbitrary units.

Supplementary Figure 5

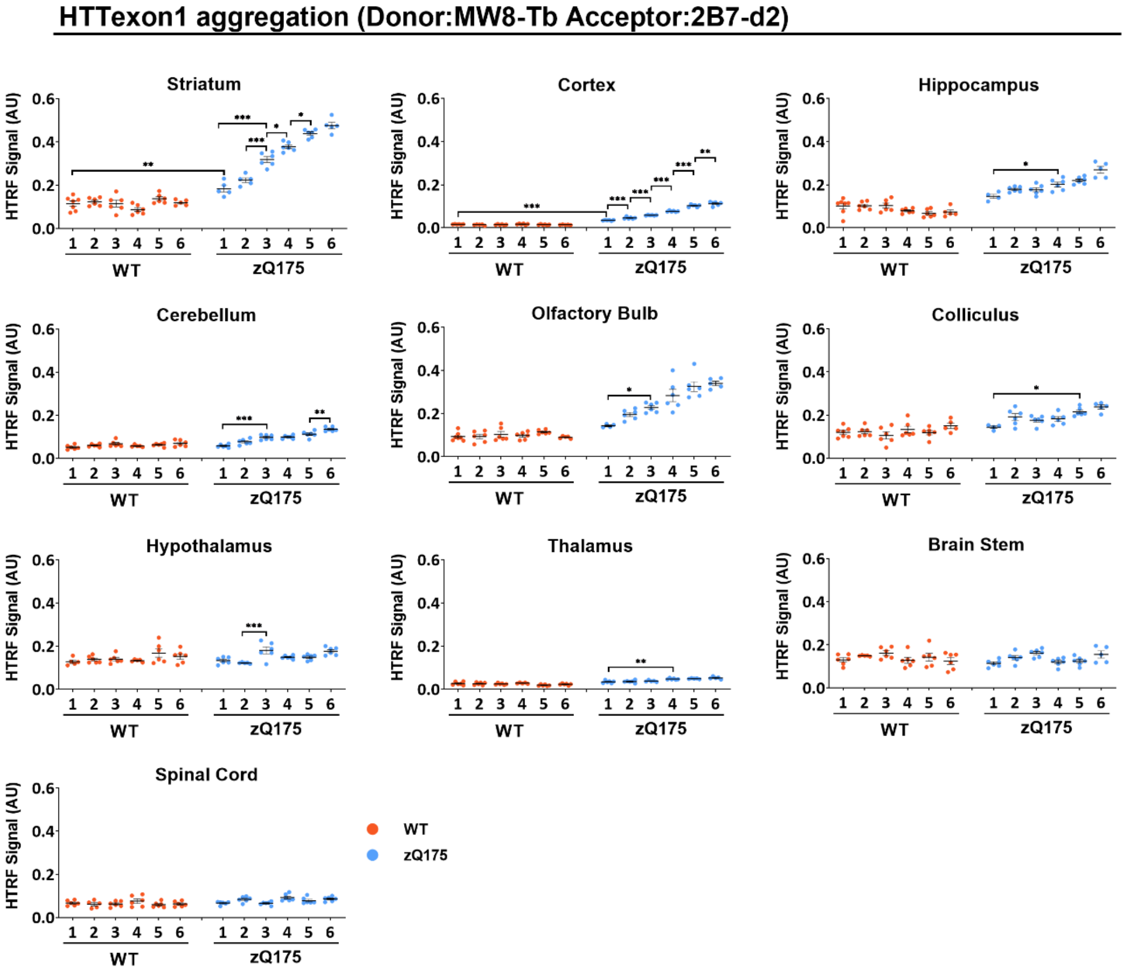

Supplementary Figure 5. Comparative increase in aggregated HTTexon1 in ten CNS regions from zQ175 mice aged from 1 – 6 months. Aggregated HTTexon1, as detected by the MW8-2B7 assay increased in all brain regions from 1 – 6 months of age except brain stem and spinal cord. The presence of aggregated HTTexon1 could be detected above background in the striatum and cortex at 1 month. The greatest level of aggregated HTTexon1 was in the striatum followed by the olfactory bulb, with none detected in the hypothalamus, thalamus, brain stem and spinal cord. N = 6 / genotype / age. Error bars are mean ± SEM. The test statistic, degrees of freedom and *p* values for the two-way ANOVA are provided in Supplementary Table 5. Statistical differences are indicated when there is a difference from one month to the next and to indicate the age at which aggregated HTTexon1 could first be detected *$p \leq 0.05$, **$p \leq 0.01$, ***$p \leq 0.001$. WT = wild type, AU = arbitrary units.

Supplementary Figure 6

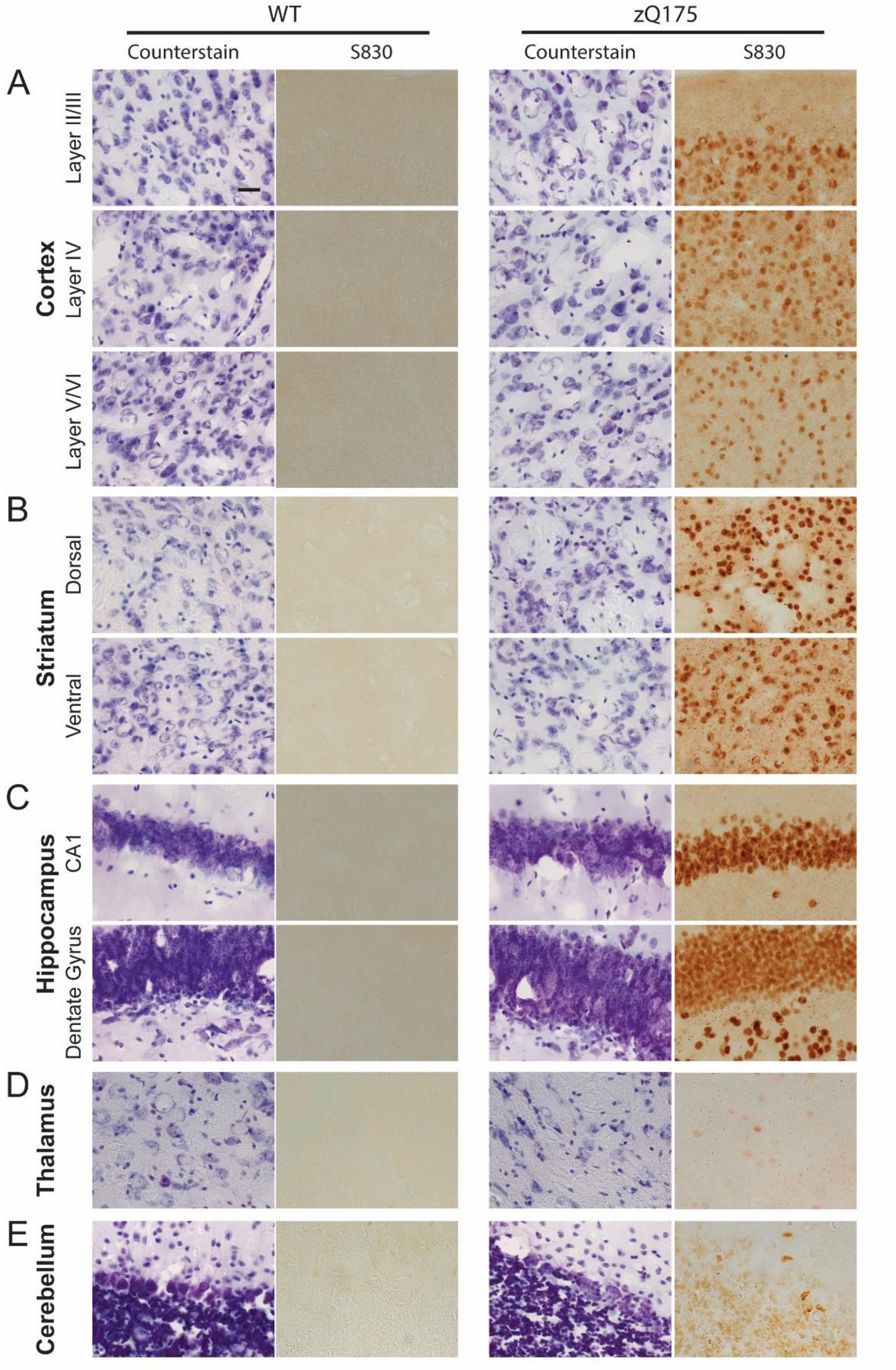

Supplementary Figure 6. Coronal sections from wild-type and zQ175 mice counterstained with cresol violet. Coronal sections from 6-month-old zQ175 and wild-type mice were counterstained with cresol violet to show the location of cell nuclei. These are shown adjacent to the comparable brain region that has been immunostained with S830. WT = wild type. N = 3. Scale bar = 20 µm.

Supplementary Figure 7

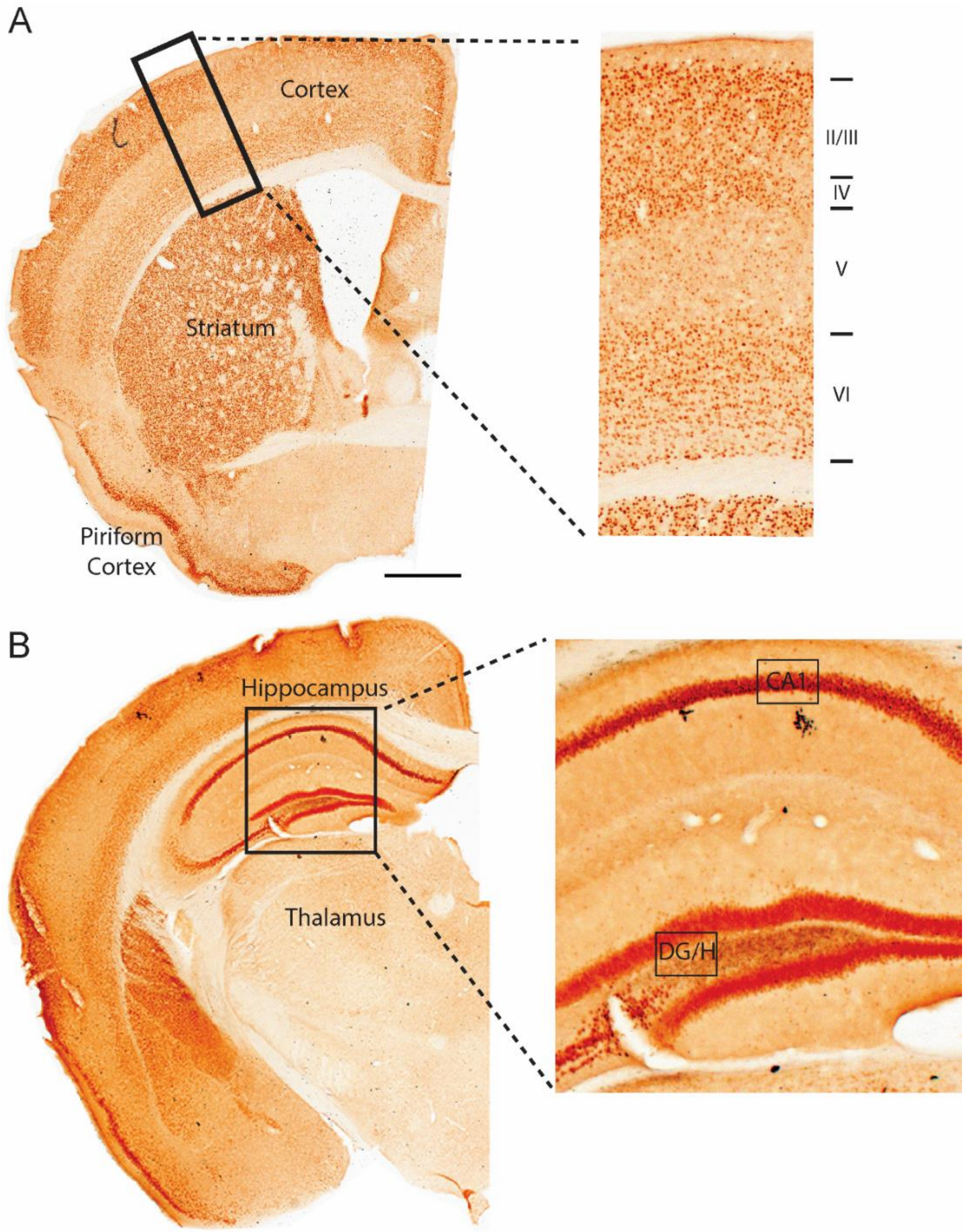

Supplementary Figure 7. HTT aggregation in the brains of zQ175 mice. Coronal sections from the brains of 6-month-old zQ175 mice were immunoprobed with S830 at (**A**) the level of the striatum and (**B**) the level of the hippocampus. The boxes in (A) and (B) indicate the location from which the higher power images in Figs. 5 and 6 were taken. N = 3. Scale bar = 1mm. DG = dentate gyrus, H = hilus.

Supplementary Figure 8

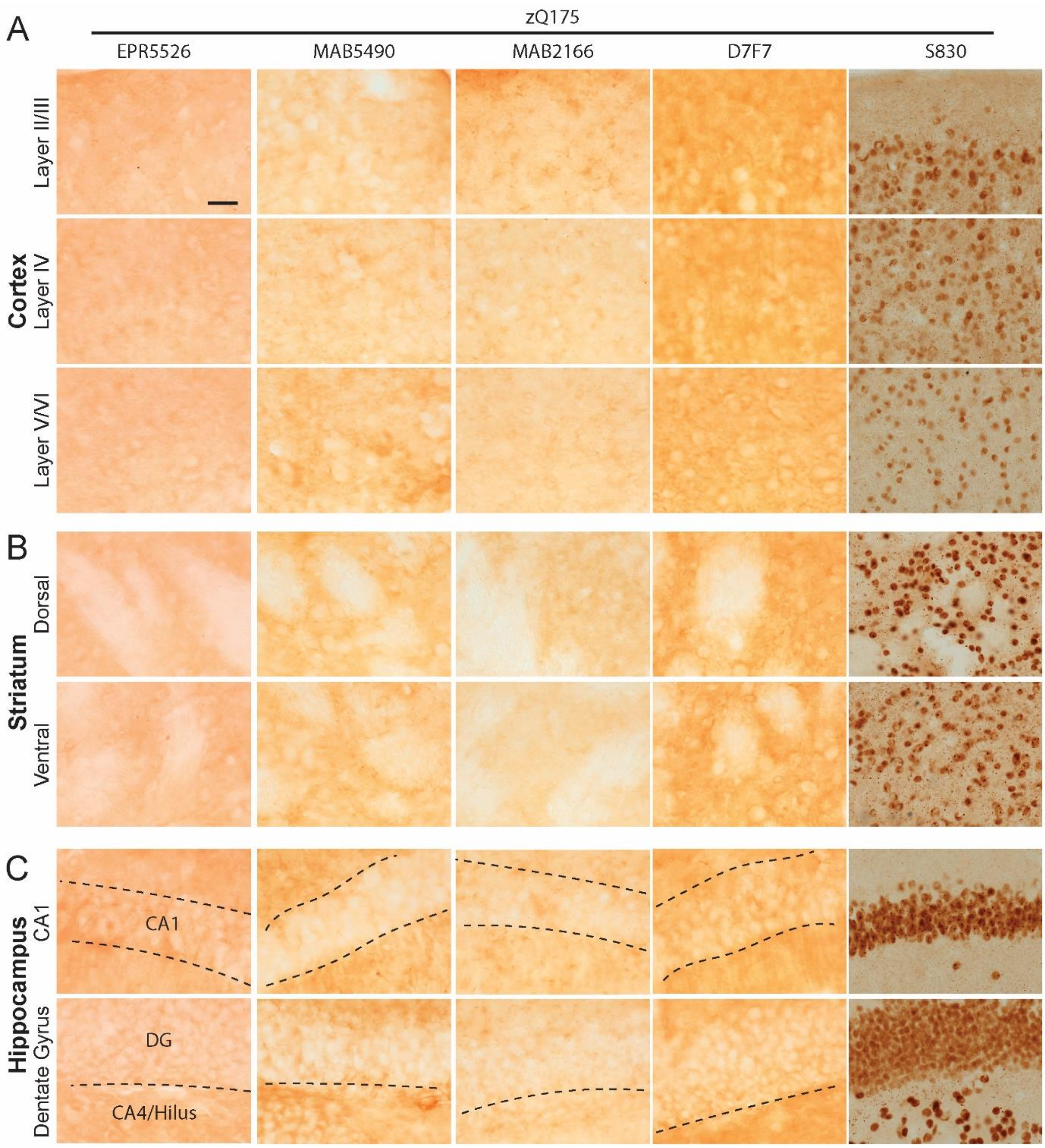

Supplementary Figure 8. HTT aggregates were not detected by antibodies binding to the first 17 amino acids of HTT or to epitopes C-terminal to HTTexon1. Coronal sections from zQ175 and wild-type mice at 6 months of age were immunoprobed with EPR5526 (1 – 17 amino acids) and MAB5490 (115 - 129), MAB2166 (443 – 457) and D7F7 (around Pro1220). In contrast to S830, there was no staining above wild-type levels with any of these antibodies in the cortex, striatum or hippocampus. N = 3. Scale bar = 20 µm.

Supplementary Figure 9

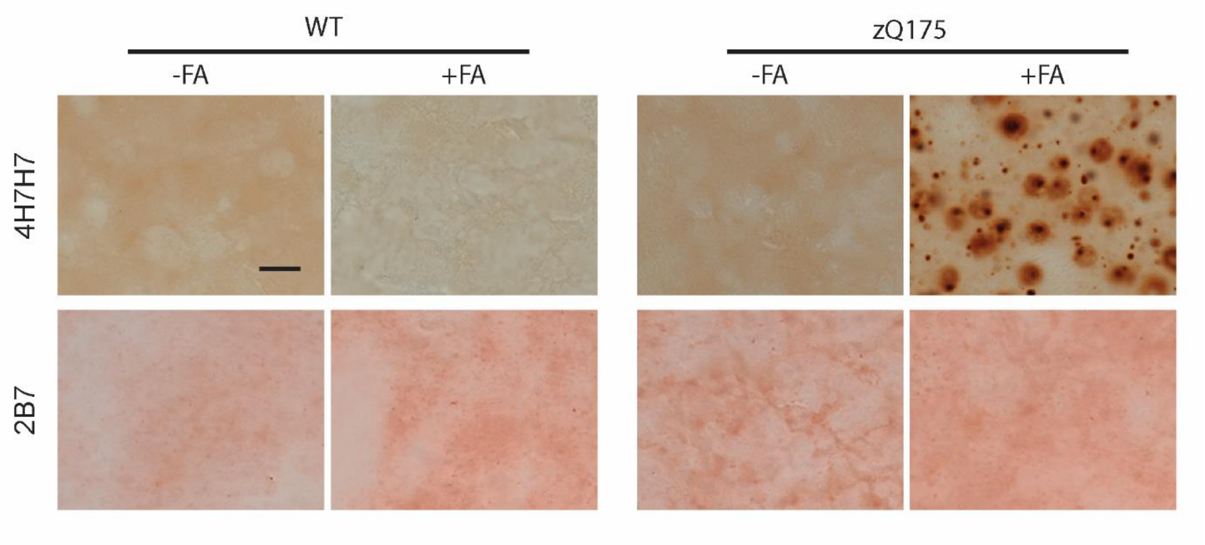

Supplementary Figure 9. The 2B7 epitope was not exposed by antigen retrieval with formic acid. Immunohistochemistry with the 2B7 and 4H7H7 antibodies to striatal sections from zQ175 and wild-type mice at 6 months of age that had, or had not, been pretreated with formic acid. Neither 4H7H7 nor 2B7 detected HTT aggregates on zQ175 sections without formic acid treatment. Antigen retrieval with formic acid exposed the polyglutamine 4H7H7 epitope, but not that for 2B7. Aggregates were only detected with 4H7H7. No staining was observed in the wild-type controls. Scale bar = 5 µm. WT = wild type, FA = formic acid.

Supplementary Figure 10

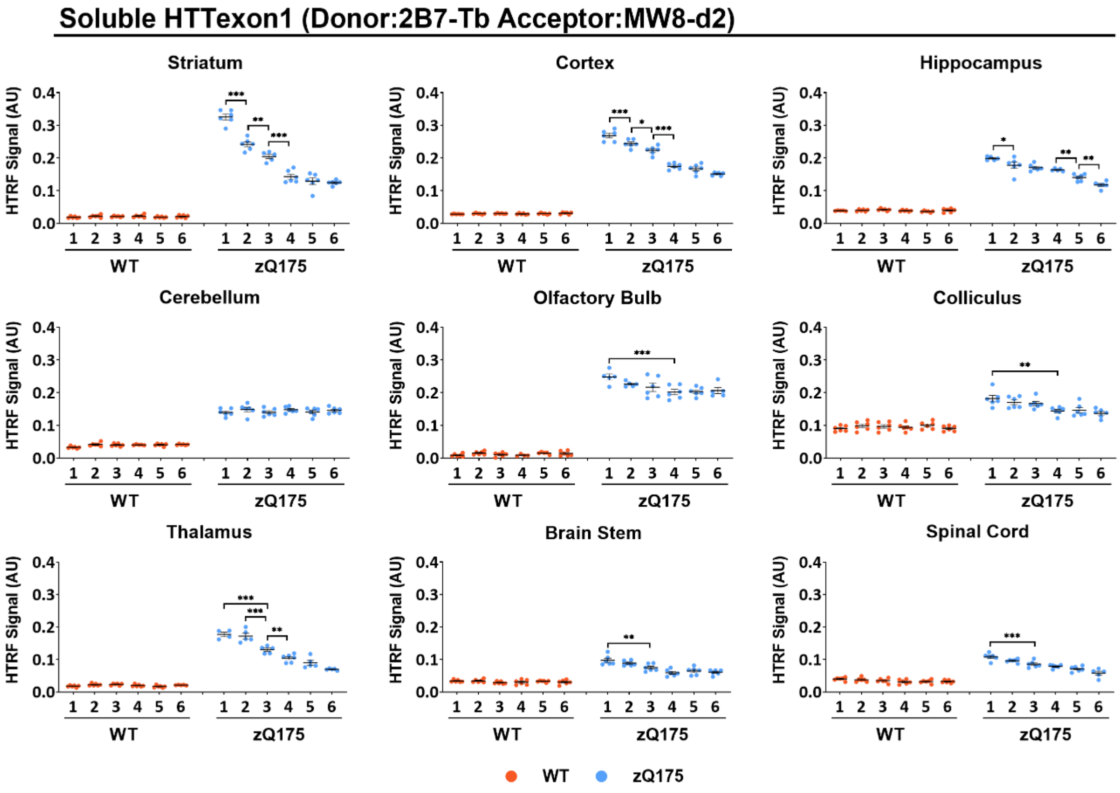

Supplementary Figure 10. Comparative decrease in soluble HTTexon1 levels in nine CNS regions from zQ175 mice aged from 1 – 6 months. The level of soluble HTTexon1 as detected by the 2B7-MW8 HTRF assay decreased in all brain regions from 1 – 6 months of age except for cerebellum. The brain regions with the highest levels of HTTexon1 were broadly comparable to those with the highest levels of HTT aggregation, as measured by HTRF. N = 6 / genotype / age. Error bars are mean ± SEM. The test statistic, degrees of freedom and *p* values for the two-way ANOVA are provided in Supplementary Table 8. Statistical differences are indicated when there is a difference from one month to the next and to indicate the age at which the level of soluble HTTexon1 were first found to decrease *$p \leq 0.05$, **$p \leq 0.01$, ***$p \leq 0.001$. WT = wild type, AU = arbitrary units.

Supplementary Figure 11

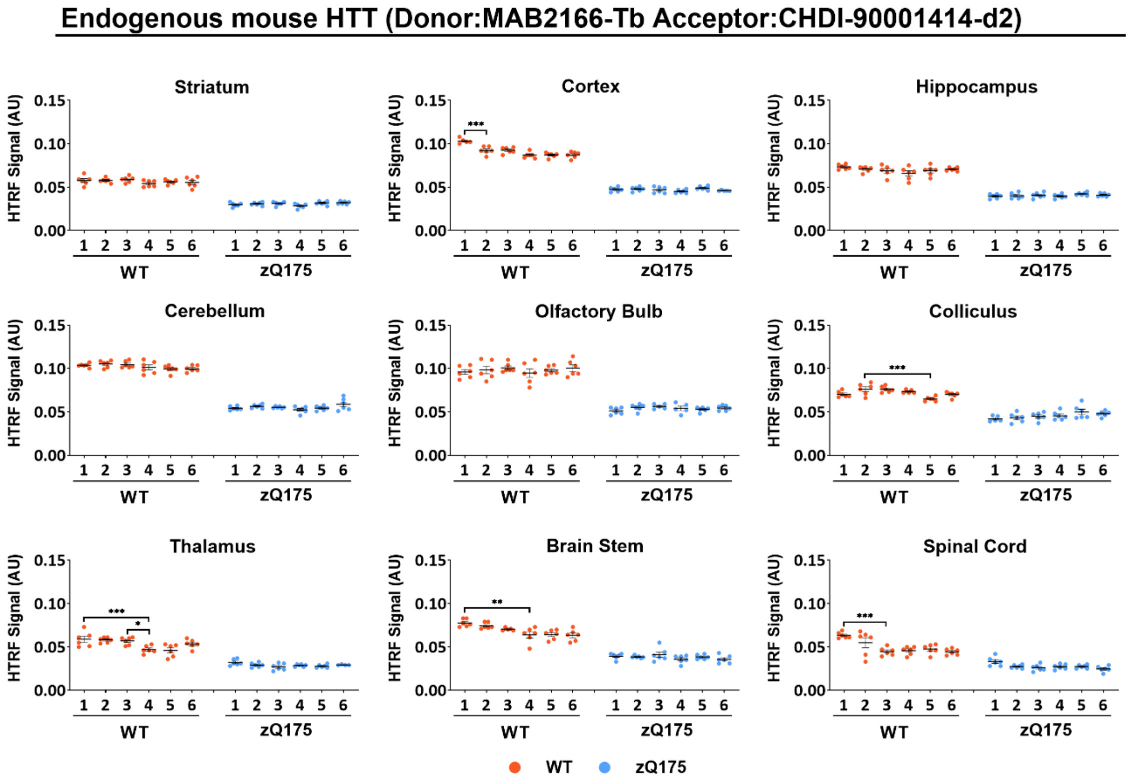

Supplementary Figure 11. Comparative level of endogenous mouse HTT in nine CNS regions from zQ175 and wild-type mice aged from 1 – 6 months. Endogenous mouse HTT was detected using the MAB2166-CHDI-1414 assay. The levels were higher in wild-type mice that have two copies of endogenous mouse HTT, compared to zQ175 mice that have one copy. Endogenous mouse HTT levels did not change in zQ175 mice over the 6-month period in any brain region. There was some variability in HTT levels in five brain regions in wild-type mice, but overall, the levels were stable. N = 6 / genotype / age. Error bars are mean ± SEM. The test statistic, degrees of freedom and *p* values for the two-way ANOVA are provided in Supplementary Table 9. Statistical differences indicate the age at which levels in endogenous mouse HTT had first changed $^{*}p \leq 0.05$, $^{**}p \leq 0.01$, $^{***}p \leq 0.001$. WT = wild type, AU = arbitrary units.

Supplementary Figure 12

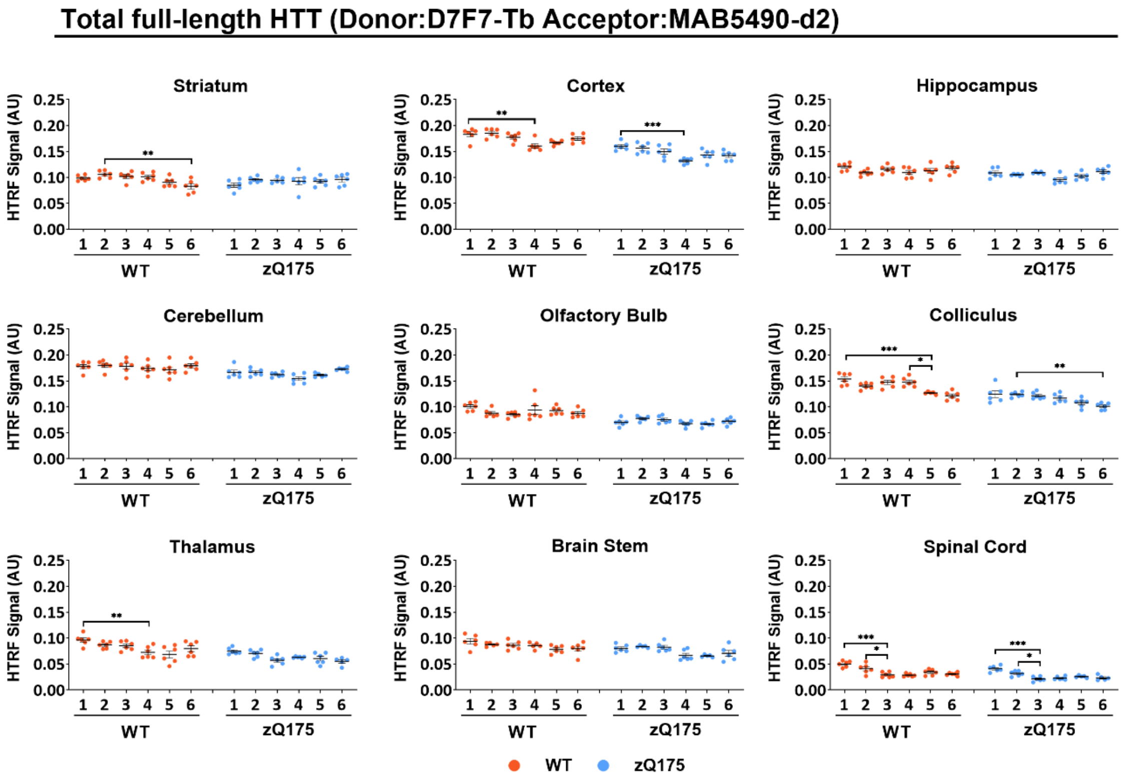

Supplementary Figure 12. Comparative level of total full-length HTT in nine CNS regions from zQ175 and wild-type mice aged from 1 – 6 months. Total full-length HTT (mutant and wild-type) was detected using the D7F7-MAB5490 assay. The levels of total full-length HTT were comparable between wild-type and zQ175 mice. There was no consistent change in total HTT levels in zQ175 mice between brain regions. In the cortex, colliculus and spinal cord, there was a comparable decrease in total HTT levels in wild-type and zQ175 mice over the six-month period, suggesting that this is not due to the influence of the HTT mutation. Overall, the levels of total HTT were stable. N = 6 / genotype / age. Error bars are mean ± SEM. The test statistic, degrees of freedom and *p* values for the two-way ANOVA are provided in Supplementary Table 10. Statistical differences indicate the age at which levels in total full-length HTT had first be found to change **p* ≤ 0.05, ***p* ≤ 0.01, ****p* ≤ 0.001. WT = wild type, AU = arbitrary units.

Supplementary Figure 13

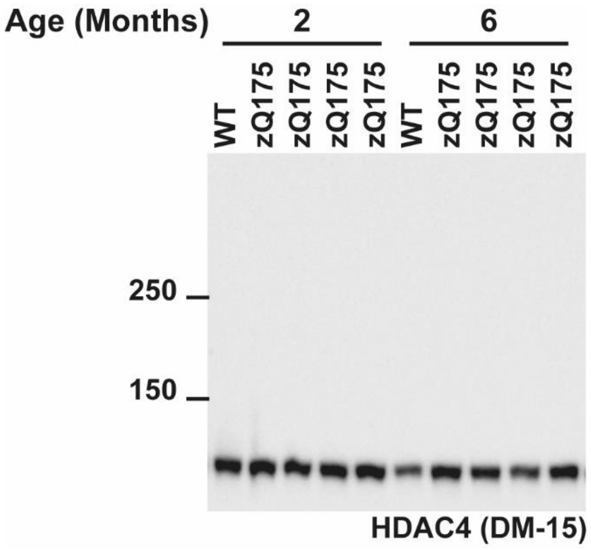

Supplementary Figure 13. Full-sized loading control blot. The full-sized loading control blot immunoprobed with HDAC4 for the western blot presented in Fig. 9C. The molecular weight markers are in kDa. WT = wild type.

Supplementary Table 3. The test statistic, degrees of freedom and *p* values for the two-way ANOVA of data presented in Fig. 2A and Supplementary Fig. 3.

	**Donor Ab: 4C9-Tb Acceptor Ab: S830-d2**			
	Striatum		Cortex	
Age x Genotype	F (5, 59) = 102.0	P<0.001	F (5, 57) = 608.4	P<0.001
Age	F (5, 59) = 111.5	P<0.001	F (5, 57) = 612.1	P<0.001
Genotype	F (1, 59) = 2097	P<0.001	F (1, 57) = 13637	P<0.001
	Hippocampus		Cerebellum	
Age x Genotype	F (5, 60) = 141.8	P<0.001	F (5, 59) = 65.17	P<0.001
Age	F (5, 60) = 161.8	P<0.001	F (5, 59) = 87.25	P<0.001
Genotype	F (1, 60) = 3064	P<0.001	F (1, 59) = 1660	P<0.001
	Olfactory Bulb		Colliculus	
Age x Genotype	F (5, 60) = 84.01	P<0.001	F (5, 56) = 28.47	P<0.001
Age	F (5, 60) = 79.39	P<0.001	F (5, 56) = 14.54	P<0.001
Genotype	F (1, 60) = 2241	P<0.001	F (1, 56) = 423.8	P<0.001
	Hypothalamus		Thalamus	
Age x Genotype	F (5, 57) = 85.31	P<0.001	F (5, 57) = 87.54	P<0.001
Age	F (5, 57) = 104.1	P<0.001	F (5, 57) = 79.85	P<0.001
Genotype	F (1, 57) = 3064	P<0.001	F (1, 57) = 1987	P<0.001
	Brain Stem		Spinal Cord	
Age x Genotype	F (5, 57) = 63.01	P<0.001	F (5, 56) = 50.25	P<0.001
Age	F (5, 57) = 66.11	P<0.001	F (5, 56) = 49.39	P<0.001
Genotype	F (1, 57) = 842.4	P<0.001	F (1, 56) = 831.0	P<0.001

Supplementary Table 4. The test statistic, degrees of freedom and *p* values for the two-way ANOVA of data presented in Fig. 2B and Supplementary Fig. 4.

	**Donor Ab: 4C9-Tb Acceptor Ab: MW8-d2**			
	Striatum		Cortex	
Age x Genotype	F (5, 56) = 712.2	P<0.001	F (5, 59) = 410.7	P<0.001
Age	F (5, 56) = 706.4	P<0.001	F (5, 59) = 385.0	P<0.001
Genotype	F (1, 56) = 7964	P<0.001	F (1, 59) = 3400	P<0.001
	Hippocampus		Cerebellum	
Age x Genotype	F (5, 56) = 409.1	P<0.001	F (5, 60) = 280.5	P<0.001
Age	F (5, 56) = 389.5	P<0.001	F (5, 60) = 310.0	P<0.001
Genotype	F (1, 56) = 3579	P<0.001	F (1, 60) = 2862	P<0.001
	Olfactory Bulb		Colliculus	
Age x Genotype	F (5, 55) = 325.9	P<0.001	F (5, 56) = 192.3	P<0.001
Age	F (5, 55) = 316.1	P<0.001	F (5, 56) = 203.1	P<0.001
Genotype	F (1, 55) = 3517	P<0.001	F (1, 56) = 2155	P<0.001
	Hypothalamus		Thalamus	
Age x Genotype	F (5, 57) = 21.65	P<0.001	F (5, 57) = 253.3	P<0.001
Age	F (5, 57) = 34.21	P<0.001	F (5, 57) = 238.7	P<0.001
Genotype	F (1, 57) = 551.5	P<0.001	F (1, 57) = 2711	P<0.001
	Brain Stem		Spinal Cord	
Age x Genotype	F (5, 59) = 125.2	P<0.001	F (5, 58) = 127.9	P<0.001
Age	F (5, 59) = 118.6	P<0.001	F (5, 58) = 126.0	P<0.001
Genotype	F (1, 59) = 1269	P<0.001	F (1, 58) = 1275	P<0.001

Supplementary Table 5. The test statistic, degrees of freedom and *p* values for the two-way ANOVA of data presented in Fig. 2C and Supplementary Fig. 5.

	**Donor Ab: MW8-Tb Acceptor Ab: 2B7-d2**			
	Striatum		Cortex	
Age x Genotype	F (5, 57) = 53.29	P<0.001	F (5, 58) = 185.7	P<0.001
Age	F (5, 57) = 55.97	P<0.001	F (5, 58) = 178.0	P<0.001
Genotype	F (1, 57) = 1165	P<0.001	F (1, 58) = 3682	P<0.001
	Hippocampus		Cerebellum	
Age x Genotype	F (5, 57) = 15.16	P<0.001	F (5, 59) = 11.73	P<0.001
Age	F (5, 57) = 4.326	P=0.002	F (5, 59) = 25.98	P<0.001
Genotype	F (1, 57) = 371.5	P<0.001	F (1, 59) = 168.0	P<0.001
	Olfactory Bulb		Colliculus	
Age x Genotype	F (5, 57) = 13.97	P<0.001	F (5, 57) = 2.502	P=0.04
Age	F (5, 57) = 16.64	P<0.001	F (5, 57) = 6.323	P<0.001
Genotype	F (1, 57) = 380.5	P<0.001	F (1, 57) = 97.06	P<0.001
	Hypothalamus		Thalamus	
Age x Genotype	F (5, 57) = 2.899	P=0.02	F (5, 56) = 11.24	P<0.001
Age	F (5, 57) = 4.911	P<0.001	F (5, 56) = 5.320	P<0.001
Genotype	F (1, 57) = 2.159	P=0.15	F (1, 56) = 234.6	P<0.001
	Brain Stem		Spinal Cord	
Age x Genotype	F (5, 58) = 1.176	P=0.33	F (5, 59) = 1.507	P=0.20
Age	F (5, 58) = 3.358	P=0.010	F (5, 59) = 3.083	P=0.02
Genotype	F (1, 58) = 0.2845	P=0.60	F (1, 59) = 17.46	P<0.001

Supplementary Table 6. The test statistic, degrees of freedom and *p* values for the two-way ANOVA of data presented in Fig. 2D.

	**FRASE**			
	Striatum		Cortex	
Age x Genotype	F (3, 24) = 12.34	P<0.001	F (3, 24) = 26.96	P<0.001
Age	F (3, 24) = 11.72	P<0.001	F (3, 24) = 25.74	P<0.001
Genotype	F (1, 24) = 46.57	P<0.001	F (1, 24) = 62.52	P<0.001
	Hippocampus		Cerebellum	
Age x Genotype	F (3, 24) = 15.86	P<0.001	F (3, 24) = 33.85	P<0.001
Age	F (3, 24) = 10.08	P<0.001	F (3, 24) = 30.80	P<0.001
Genotype	F (1, 24) = 39.60	P<0.001	F (1, 24) = 152.3	P<0.001

Supplementary Table 7. The test statistic, degrees of freedom and *p* values for the two-way ANOVA of data presented in Fig. 3.

	**Donor Ab: 2B7-Tb Acceptor Ab: MW1-d2**			
	zQ175		N171-82Q	
Age x Genotype	F (2, 16) = 181.5	P<0.0001	F (1, 12) = 53.24	P<0.0001
Age	F (2, 16) = 181.5	P<0.0001	F (1, 12) = 54.63	P<0.0001
Genotype	F (1, 16) = 3054	P<0.0001	F (1, 12) = 6311	P<0.0001
	**Donor Ab: 2B7-Tb Acceptor Ab: MW8-d2**			
	zQ175		N171-82Q	
Age x Genotype	F (2, 18) = 69.59	P<0.0001	F (1, 12) = 1.070	P=0.3214
Age	F (2, 18) = 67.75	P<0.0001	F (1, 12) = 1.927	P=0.1903
Genotype	F (1, 18) = 1017	P<0.0001	F (1, 12) = 0.9534	P=0.3481
	**Donor Ab: 4C9-Tb Acceptor Ab: S830-d2**			
	zQ175		N171-82Q	
Age x Genotype	F (2, 18) = 560.3	P<0.0001	F (1, 12) = 5.379	P=0.0388
Age	F (2, 18) = 563.0	P<0.0001	F (1, 12) = 6.308	P=0.0273
Genotype	F (1, 18) = 3345	P<0.0001	F (1, 12) = 73.32	P<0.0001
	**Donor Ab: 4C9-Tb Acceptor Ab: MW8-d2**			
	zQ175		N171-82Q	
Age x Genotype	F (2, 18) = 404.2	P<0.0001	F (1, 11) = 3.106	P=0.1057
Age	F (2, 18) = 408.2	P<0.0001	F (1, 11) = 37.67	P<0.0001
Genotype	F (1, 18) = 1692	P<0.0001	F (1, 11) = 0.7488	P=0.4053
	**Donor Ab: MW8-Tb Acceptor Ab: 2B7-d2**			
	zQ175		N171-82Q	
Age x Genotype	F (2, 18) = 32.96	P<0.0001	F (1, 12) = 2.194	P=0.1643
Age	F (2, 18) = 35.19	P<0.0001	F (1, 12) = 2.593	P=0.1333
Genotype	F (1, 18) = 1074	P<0.0001	F (1, 12) = 12.73	P=0.0039

Supplementary Table 8. The test statistic, degrees of freedom and *p* values for the two-way ANOVA of data presented in Supplementary Fig. 10.

	**Donor Ab: 2B7-Tb Acceptor Ab: MW8-d2**			
	Striatum		Cortex	
Age x Genotype	F (5, 59) = 110.8	P<0.001	F (5, 60) = 86.37	P<0.001
Age	F (5, 59) = 108.0	P<0.001	F (5, 60) = 82.58	P<0.001
Genotype	F (1, 59) = 3122	P<0.001	F (1, 60) = 6969	P<0.001
	Hippocampus		Cerebellum	
Age x Genotype	F (5, 57) = 26.56	P<0.001	F (5, 59) = 0.4240	P=0.83
Age	F (5, 57) = 28.08	P<0.001	F (5, 59) = 1.560	P=0.19
Genotype	F (1, 57) = 3000	P<0.001	F (1, 59) = 2369	P<0.001
	Olfactory Bulb		Colliculus	
Age x Genotype	F (5, 54) = 4.267	P=0.002	F (5, 60) = 4.669	P=0.001
Age	F (5, 54) = 3.381	P=0.010	F (5, 60) = 4.468	P=0.002
Genotype	F (1, 54) = 2998	P<0.001	F (1, 60) = 323.4	P<0.001
	Thalamus		Brain Stem	
Age x Genotype	F (5, 53) = 55.85	P<0.001	F (5, 60) = 8.278	P<0.001
Age	F (5, 53) = 57.91	P<0.001	F (5, 60) = 12.31	P<0.001
Genotype	F (1, 53) = 1949	P<0.001	F (1, 60) = 439.4	P<0.001
	Spinal Cord			
Age x Genotype	F (5, 59) = 10.53	P<0.001		
Age	F (5, 59) = 21.14	P<0.001		
Genotype	F (1, 59) = 682.7	P<0.001		

Supplementary Table 9. The test statistic, degrees of freedom and *p* values for the two-way ANOVA of data presented in Supplementary Fig. 11.

	**Donor Ab: MAB2166-Tb Acceptor Ab: CHDI-90001414-d2**			
	Striatum		Cortex	
Age x Genotype	F (5, 60) = 0.8841	P=0.50	F (5, 58) = 11.53	P<0.001
Age	F (5, 60) = 1.889	P=0.11	F (5, 58) = 13.49	P<0.001
Genotype	F (1, 60) = 1133	P<0.001	F (1, 58) = 3698	P<0.001
	Hippocampus		Cerebellum	
Age x Genotype	F (5, 60) = 1.523	P=0.20	F (5, 60) = 1.956	P=0.10
Age	F (5, 60) = 1.331	P=0.26	F (5, 60) = 1.828	P=0.12
Genotype	F (1, 60) = 996.2	P<0.001	F (1, 60) = 2358	P<0.001
	Olfactory Bulb		Colliculus	
Age x Genotype	F (5, 58) = 0.2174	P=0.95	F (5, 58) = 6.336	P<0.001
Age	F (5, 58) = 0.9055	P=0.48	F (5, 58) = 1.392	P=0.24
Genotype	F (1, 58) = 756.3	P<0.001	F (1, 58) = 574.7	P<0.001
	Thalamus		Brain Stem	
Age x Genotype	F (5, 59) = 3.789	P=0.005	F (5, 59) = 2.967	P=0.02
Age	F (5, 59) = 6.321	P<0.001	F (5, 59) = 6.625	P<0.001
Genotype	F (1, 59) = 514.2	P<0.001	F (1, 59) = 644.2	P<0.001
	Spinal Cord			
Age x Genotype	F (5, 60) = 2.458	P=0.04		
Age	F (5, 60) = 10.19	P<0.001		
Genotype	F (1, 60) = 290.4	P<0.001		

Supplementary Table 10. The test statistic, degrees of freedom and *p* values for the two-way ANOVA of data presented in Supplementary Fig. 12.

	**Donor Ab: D7F7-Tb Acceptor Ab: MAB5490-d2**			
	Striatum		Cortex	
Age x Genotype	F (5, 59) = 3.105	P=0.01	F (5, 60) = 0.4687	P=0.80
Age	F (5, 59) = 2.530	P=0.04	F (5, 60) = 13.36	P<0.001
Genotype	F (1, 59) = 3.374	P=0.07	F (1, 60) = 160.9	P<0.001
	Hippocampus		Cerebellum	
Age x Genotype	F (5, 59) = 0.7012	P=0.62	F (5, 59) = 0.7311	P=0.60
Age	F (5, 59) = 4.508	P=0.002	F (5, 59) = 2.495	P=0.04
Genotype	F (1, 59) = 22.24	P<0.001	F (1, 59) = 33.02	P<0.001
	Olfactory Bulb		Colliculus	
Age x Genotype	F (5, 60) = 3.088	P=0.02	F (5, 59) = 1.350	P=0.26
Age	F (5, 60) = 0.8113	P=0.55	F (5, 59) = 17.63	P<0.001
Genotype	F (1, 60) = 90.42	P<0.001	F (1, 59) = 125.0	P<0.001
	Thalamus		Brain Stem	
Age x Genotype	F (5, 59) = 2.023	P=0.09	F (5, 56) = 1.218	P=0.31
Age	F (5, 59) = 7.972	P<0.001	F (5, 56) = 5.818	P<0.001
Genotype	F (1, 59) = 59.15	P<0.001	F (1, 56) = 22.38	P<0.001
	Spinal Cord			
Age x Genotype	F (5, 58) = 0.1962	P=0.96		
Age	F (5, 58) = 28.37	P<0.001		
Genotype	F (1, 58) = 41.02	P<0.001		

REFERENCES

1. Weiss A, Abramowski D, Bibel M*, et al*. Single-step detection of mutant huntingtin in animal and human tissues: A bioassay for Huntington's disease. *Anal Biochem*. Dec 1 2009;395(1):8-15.

2. Ko J, Ou S, Patterson PH. New anti-huntingtin monoclonal antibodies: implications for huntingtin conformation and its binding proteins. *Brain Res Bull*. Oct-Nov 1 2001;56(3-4):319-329.

3. Landles C, Sathasivam K, Weiss A*, et al*. Proteolysis of mutant huntingtin produces an exon 1 fragment that accumulates as an aggregated protein in neuronal nuclei in Huntington disease. *J Biol Chem*. Mar 19 2010;285(12):8808-8823.

4. Sathasivam K, Woodman B, Mahal A*, et al*. Centrosome disorganization in fibroblast cultures derived from R6/2 Huntington's disease (HD) transgenic mice and HD patients. *Hum Mol Genet*. Oct 1 2001;10(21):2425-35.

5. Cong SY, Pepers BA, Roos RA, Van Ommen GJ, Dorsman JC. Epitope mapping of monoclonal antibody 4C8 recognizing the protein huntingtin. *Hybridoma (Larchmt)*. Oct 2005;24(5):231-5.
